# Protected Area Tourism in a Changing Climate: Will Visitation at US National Parks Warm Up or Overheat?

**DOI:** 10.1371/journal.pone.0128226

**Published:** 2015-06-17

**Authors:** Nicholas A. Fisichelli, Gregor W. Schuurman, William B. Monahan, Pamela S. Ziesler

**Affiliations:** Natural Resource Science and Stewardship, US National Park Service, Fort Collins, Colorado, United States of America; U.S. Geological Survey, UNITED STATES

## Abstract

Climate change will affect not only natural and cultural resources within protected areas but also tourism and visitation patterns. The U.S. National Park Service systematically collects data regarding its 270+ million annual recreation visits, and therefore provides an opportunity to examine how human visitation may respond to climate change from the tropics to the polar regions. To assess the relationship between climate and park visitation, we evaluated historical monthly mean air temperature and visitation data (1979–2013) at 340 parks and projected potential future visitation (2041–2060) based on two warming-climate scenarios and two visitation-growth scenarios. For the entire park system a third-order polynomial temperature model explained 69% of the variation in historical visitation trends. Visitation generally increased with increasing average monthly temperature, but decreased strongly with temperatures > 25°C. Linear to polynomial monthly temperature models also explained historical visitation at individual parks (R^2^ 0.12-0.99, mean = 0.79, median = 0.87). Future visitation at almost all parks (95%) may change based on historical temperature, historical visitation, and future temperature projections. Warming-mediated increases in potential visitation are projected for most months in most parks (67–77% of months; range across future scenarios), resulting in future increases in total annual visits across the park system (8–23%) and expansion of the visitation season at individual parks (13–31 days). Although very warm months at some parks may see decreases in future visitation, this potential change represents a relatively small proportion of visitation across the national park system. A changing climate is likely to have cascading and complex effects on protected area visitation, management, and local economies. Results suggest that protected areas and neighboring communities that develop adaptation strategies for these changes may be able to both capitalize on opportunities and minimize detriment related to changing visitation.

## Introduction

Earth’s protected areas include over 100,000 sites [1], and climate change will affect not only biodiversity and cultural resources within these protected areas, but also human visitation. With continued warming, climatically suitable areas for tourism may move poleward and low-latitude destinations may see shifts in visitation timing as tourists avoid extremely warm periods and associated conditions [2–4]. Whether visitors track climate change and shift their behavior across protected areas will depend on multiple environmental and socioeconomic factors [3]; however, understanding potential changes in visitation based on historical trends and future patterns of temperature change is a crucial first step for protected area managers and local communities to anticipate, plan for, and proactively influence future visitation. Examining these potential changes requires an assessment of long-term visitation data across a very large system of protected areas. Here, we take advantage of the United States national park system’s diversity and abundance of protected areas, climatic breadth, and systematic visitor-use data to address this issue.

United States national parks have been called “America’s best idea” because they protect the most highly valued areas of national heritage for all people [5]. The U.S. National Park Service (NPS) serves two related roles: 1) conserve natural and cultural resources and values and 2) provide for the enjoyment, education, and inspiration of current and future generations [6]. As the NPS celebrates its centennial and enters a second century of stewardship, it recognizes that global change factors such as climate change are significant threats likely to affect both park resources and visitor use and experience [7]. Climatic conditions in an overwhelming majority of national parks are already at the warm extremes of their historical ranges of variability [8]. Furthermore, resource responses to ongoing climate change are evident across many parks and include measurable responses by glaciers, birds, insects, mammals, and vegetation [9–13]. Similar to other biota, human visitors to parks are prone to alter their behavior in response to climate change [2]. For example, peak visitor attendance appears to be shifting earlier in the year with increasing spring temperatures at some parks [14].

Multiple factors drive global tourism and protected area visitation, including financial constraints, availability of leisure time, and weather and climate [3]. Institutional schedules (e.g., national, religious, and school holidays) are also influential; nevertheless, the overall visitation pattern across protected areas worldwide consistently relates to regional climate patterns [15,16], and more specifically to direct and indirect effects of temperature [4]. Visitors to Rocky Mountain National Park in Colorado, for example, typically plan their trips more than two months in advance and thus base decisions in part on long-term climate averages [17].

Future climate change-driven shifts in visitation timing and volume will have consequences for both protected area management and local economies. The 273 million visits to the U.S. national park system in 2013 resulted in expenditures of $14.6 billion in local communities and supported over 238,000 jobs [18]. Employment opportunities within parks and local park-oriented communities change seasonally with shifts in the number of visitors. For example, short-term seasonal employees comprise, on average, over 40% of the NPS workforce at parks during peak visitation periods (NPS Office of Human Resources data). Globally, travel and tourism account for roughly 9% of the world GDP [19], and thus future climate-mediated changes to protected area tourism could have impacts beyond local communities.

The U.S. national park system includes units from the tropics to the arctic and from remote wilderness to urban settings. Continued warming may extend the visitation season and increase visitation numbers at some parks while reducing it at others. Importantly, visitation patterns at still other parks may simply be insensitive to temperature. The objective of this study was to quantify the relationship between historical visitation patterns and temperature across a large protected area system and examine potential future visitation based on this relationship and projected climate change.

## Materials and Methods

### Study sites

This study included protected areas across the U.S. national park system, from Guam and Hawai‘i in the Pacific to Alaska, the contiguous 48 states, and islands in the Caribbean. The national park system includes parks, monuments, recreation areas, historic sites, battlefields, memorials, and other designations located in urban, suburban, rural, and remote locations. These protected areas (hereafter referred to as ‘parks’) span over 150° of longitude and 80° of latitude. We used monthly total recreation visits between 1979 and 2013 for all parks with available data (*n* = 370) [20]. A recreation visit is defined as entry of a person onto lands or waters administered by the NPS for recreational purposes, excluding government personnel, through-traffic (commuters), tradespeople, and people residing within park boundaries [21]. For analyses, we included all parks with at least 10 years of data and an average of 8000 annual visits (*n* = 340) to ensure sufficient monthly visit counts, and averaged each month’s total recreation visits across all available years. Our analyses therefore capture historical long-term averages in climate and visitation and use these data and future climate projections to project potential future long-term average visitation.

### Climate data

We matched historical visitation data with gridded historical monthly mean air temperature data obtained from the Climatic Research Unit (CRU) high-resolution time series version 3.22 (TS 3.22) [22]. CRU TS 3.22 data are globally available at 0.5 decimal degrees (approximately 3000 km^2^ at the Equator and 2000 km^2^ at 70° latitude) for each month and year of visitation data. Although this dataset is spatially coarse, at least relative to the size of some parks, it offers for all NPS geographies the highest spatiotemporal resolution of observed temperature over the 1979–2013 study period.

Future monthly mean air temperature projections for each park were obtained from WorldClim [23]. We considered a 2041–2060 future and two emissions scenarios, representative concentration pathways (RCPs) 4.5 and 8.5 W/m^2^ (referenced as RCP 4.5 and 8.5). Estimates were based on 17 individual climate models available through the Coupled Model Intercomparison Project Phase 5 (CMIP5; see S1 and S2 Tables). The individual CMIP5 models were downscaled and calibrated (bias-corrected) using WorldClim as the historical (1950–2000) baseline. To account for differences between the training and projection data sources (CRU vs. WorldClim), we first subtracted each historical WorldClim monthly mean temperature variable from each corresponding WorldClim future (model × RCP). We then added these deltas to estimates of CRU monthly mean temperature computed for the same historical baseline (1950–2000). For each park we determined “low” and “high” climate change scenarios by selecting and ensemble-averaging the five lowest individual climate models under RCP 4.5, and five highest models under RCP 8.5, according to estimates of annual mean temperature (S1 and S2 Tables).

To examine historical and potential future visitation, we used historical and future monthly mean air temperature. Across parks, historical monthly mean temperature varied from—17.7°C to 33.2°C (mean = 12.6°C). For 2041–2060, projected monthly mean temperature varied from—16.7°C to 34.9°C (mean = 13.8°C) under RCP 4.5 and from—14.6°C to 36.5°C (mean = 16.0°C) under RCP 8.5. Average monthly warming under the RCP 4.5 scenario was 1.3°C and under RCP 8.5 was 3.4°C. Some tourism-climate studies use climate indices based on multiple temperature, precipitation, humidity, and other variables [24,25]. Although these climate indices may perform well, issues of multicollinearity exist and temperature is generally the most influential climate variable [4]. By using a single explanatory variable, air temperature, we assume that others factors are constant (e.g., income, population size and demography, and leisure time availability). While managers cannot influence climate change over large landscapes, they can plan and prepare for how such changes may alter visitation; knowledge of the quantitative relationship between visitation and temperature is a critical first filter for deciding where and how to possibly implement management actions.

### Statistical analyses

We parameterized system-wide and park-specific models to assess the relationship between historical visitation and climate. To examine the overall distribution of visitation and relationship to temperature across the park system, we used generalized linear models (glm) with a binomial error distribution; proportion of a park’s annual visits occurring in each month (4080 months in 340 parks) was the response variable and mean monthly temperature and park name were the explanatory variables. Using proportions of visitation for each month allowed us to compare visitation patterns across parks with strongly differing annual totals (minimum annual total = 8150 and maximum annual total = 16.8 million). We tested four models of increasing complexity (a null model [intercept only], the first-order polynomial equation of temperature, the second-order (quadratic) polynomial equation of temperature, and the third-order (cubic) polynomial equation of temperature) to quantify the relationship between visitation and temperature [26]. Park name (categorical variable) was included in all models to account for the hierarchical data structure of months within parks. Bayesian information criterion (BIC) was used to identify the best model [27] and McFadden’s R^2^ was calculated for the best model. For each individual park, we used linear regression and the same four potential models and comparison techniques as above to determine the best-fit model of historical long-term mean monthly total recreation visits.

To examine potential future visitation for the 20-year period 2041–2060, we used the best-fit model of historical visitation for each park from above and then predicted visitation based on the low and high climate change scenarios and two visitation growth ceilings (5% and 25% > mean historical visitation of the busiest month). Many parks are already challenged to accommodate more visitors during the busiest times of year (e.g., holiday weekends), and thus limits on modeled potential future increases are necessary. A maximum increase in future monthly visitation of 5% over the historical maximum month is a conservative estimate of low potential change in maximum monthly visitation over the next several decades and the 25% increase is a reasonable estimate of intermediate to high potential growth. This growth ceiling generally only limits future visitation projections for the busiest one or two months of the year (e.g., 4.1% of months across parks under the low climate change and low maximum growth scenario and 4.6% of months under the high climate change and high maximum growth scenario). Assessments of future potential changes in visitation were limited to parks with temperature as an explanatory variable in the best-fit historical model and an adjusted R^2^ ≥ 0.5.

We examined historical and projected future visitation across multiple seasons including peak season (three contiguous months with highest average visitation), shoulder seasons (two months before and two months after the peak season), and low season (three contiguous months with lowest average visitation), and assessed whether the timing (i.e., months) of potential future visitation seasons differed from the historical period. We also quantified the overall visitation season length based on the historical data and define it as beginning on the date when 10% of historical cumulative visitation is achieved and ending on the date when 10% of historical cumulative visitation remains for the year (e.g., the overall visitation season in a park that historically received 100,000 annual visits begins on the date the 10,000^th^ visit occurs and ends on the date when 10,000 visits remain for the year). To calculate overall visitation season length in days, monthly data were interpolated to daily values using a cubic spline. Data were analyzed in R (v. 3.1.1) [28].

## Results

**Fig 1:**
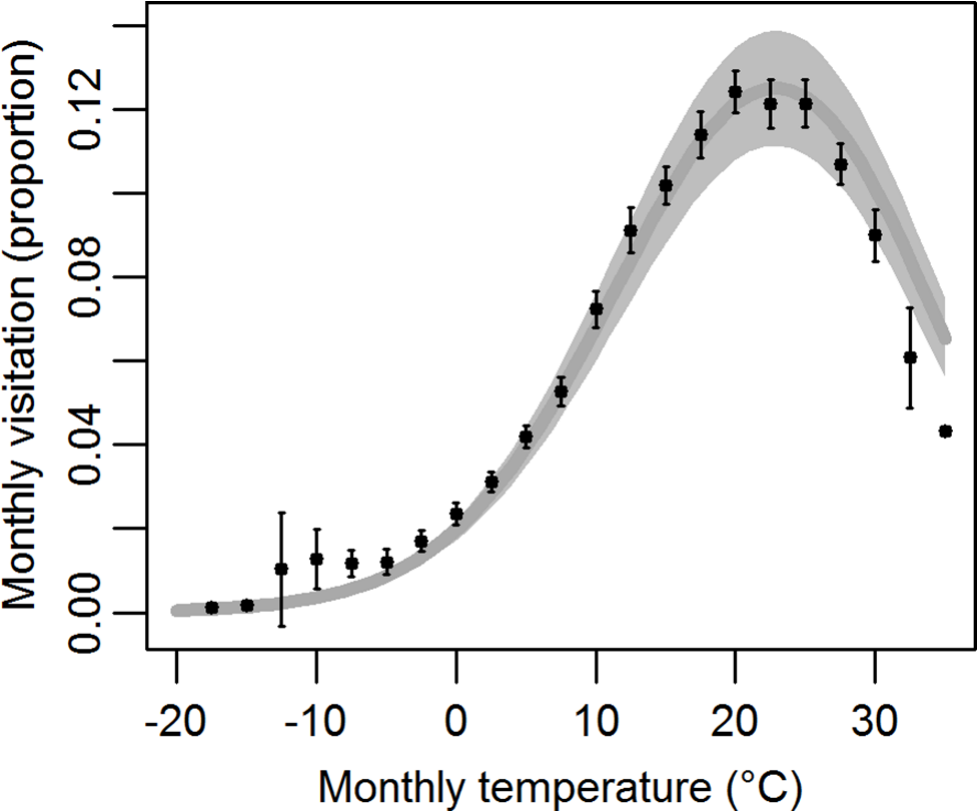
Third-order polynomial glm relationship of historical (1979–2013) monthly mean temperature and monthly park visitation (proportion of annual). Mean (black circles) and error bars (+/- twice the standard error) from observed data are based on 2.5°C bins. Model estimate (dark gray solid line) ± 1 standard error (light gray shaded area), R^2^ = 0.69.

Across the national park system, monthly historical visitation (mean proportion of annual visits) and temperature were strongly associated (Fig 1). The third-order polynomial model of temperature was the best fit (ΔBIC > 100), all temperature parameters were strongly significant (P-values ≤ 0.001), and the model had an R^2^ = 0.69 (S3 Table). This model fits the data well, though overestimates visitation at very high temperatures.

Individual parks also showed strong relationships between historical monthly mean temperature and visitation (S4 Table, S1 Fig). Temperature was included in the best-fit (lowest BIC) model for 95% of parks (324 of 340). This included the first-order model for 106 parks (31%), the second-order polynomial model for 134 parks (39%), and the third-order polynomial model for 84 parks (25%). Adjusted R^2^ for the best-fit model varied among parks from 0.12–0.99 (mean = 0.79, median = 0.87).

**Fig 2:**
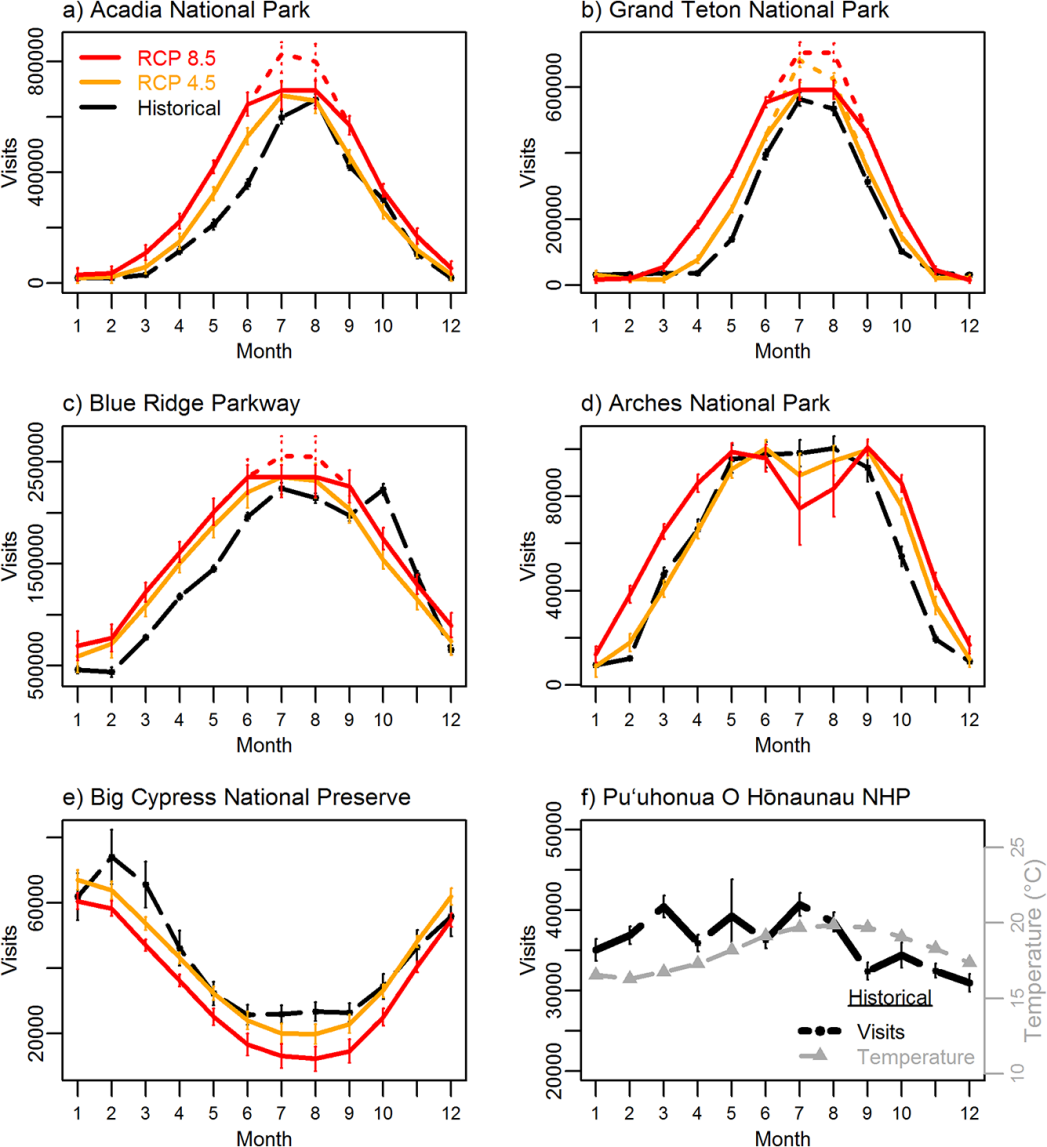
Historical (1979–2013) and potential future visitation (2041–2060) patterns in example parks. a) Acadia National Park, Maine; b) Grand Teton National Park, Wyoming; c) Blue Ridge Parkway, North Carolina and Virginia; d) Arches National Park, Utah; e) Big Cypress National Preserve, Florida; and f) Pu‘uhonua o Hōnaunau NHP (National Historical Park, Hawai‘i; where a visitation-temperature relationship was not supported and historical mean monthly temperature [gray triangles and dashed line] is shown). Historical (black) error bars are +/- one standard error and future projection error bars show +/- one standard error of the prediction estimates. Solid red lines are for RCP 8.5 and solid orange lines are for RCP 4.5 low maximum growth projections, dotted lines of the same color and thickness show high maximum growth projections when different from low maximum growth.

Of the original 340 parks assessed, 282 (83%) were temperature-sensitive, in that they showed strong relationships between visitation and temperature (adjusted R^2^ ≥ 0.5). Potential future visitation (2041–2060) varied across parks, but numerous patterns emerged (Fig 2). Many high-latitude and high-elevation parks show increases in potential visitation across most of the year and especially during the shoulder seasons (Fig 2A, 2B and 2C). Some parks with historically warm temperatures and a broad visitation peak period show a potential future decrease in visitation during the hottest months of the year (Fig 2D). Parks with already-low visitation during the hottest months show a potential for this pattern to intensify (Fig 2E). Potential future visitation patterns at many high-latitude and high-elevation parks may look more like historical visitation at mid-latitude parks (Fig 2A and 2B to Fig 2C and 2D), and patterns at mid-latitude parks under a warmer future may look more like historical patterns at southern parks (Fig 2D to Fig 2E). Other parks, including many tropical and subtropical parks with relatively narrow monthly temperature ranges, showed no relationship to temperature, and therefore no warming-driven change was supported (Fig 2F). Some busy visitation periods such as late winter/early spring in the southern U.S. (Fig 2E) and autumn foliage season in the eastern U.S. (Fig 2C) were not captured in these temperature-only models.

**Fig 3:**
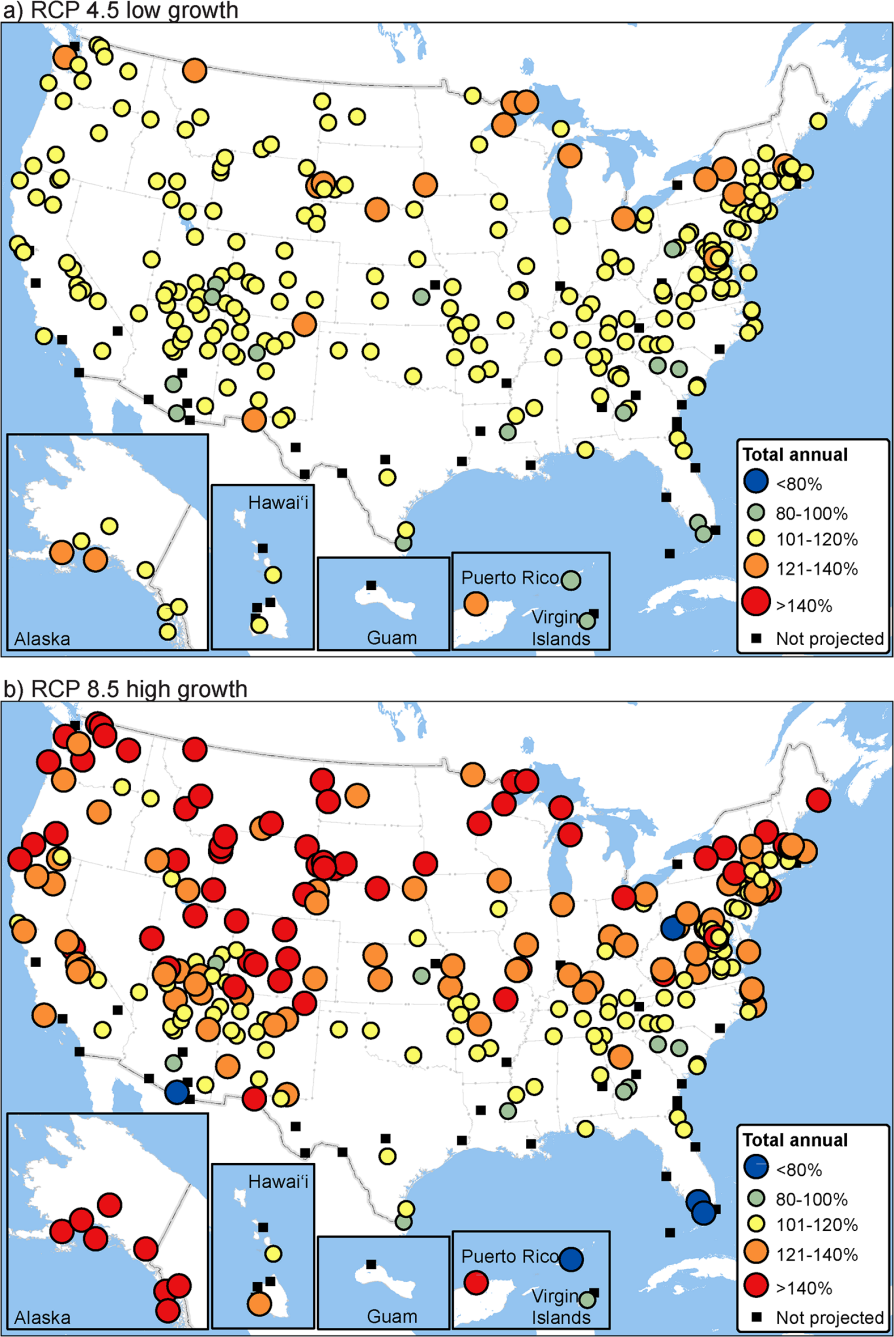
Potential future total annual visitation (2041–2060) expressed as a percentage of historical values (1979–2013). Future visitation for each park is based on modeled monthly visitation relationship to temperature. Future projections, a) RCP 4.5 low growth and b) RCP 8.5 high growth, were limited to parks with temperature as an explanatory variable in the best-fit model and an adjusted R^2^ ≥ 0.5 (*n* = 282).

**Fig 4:**
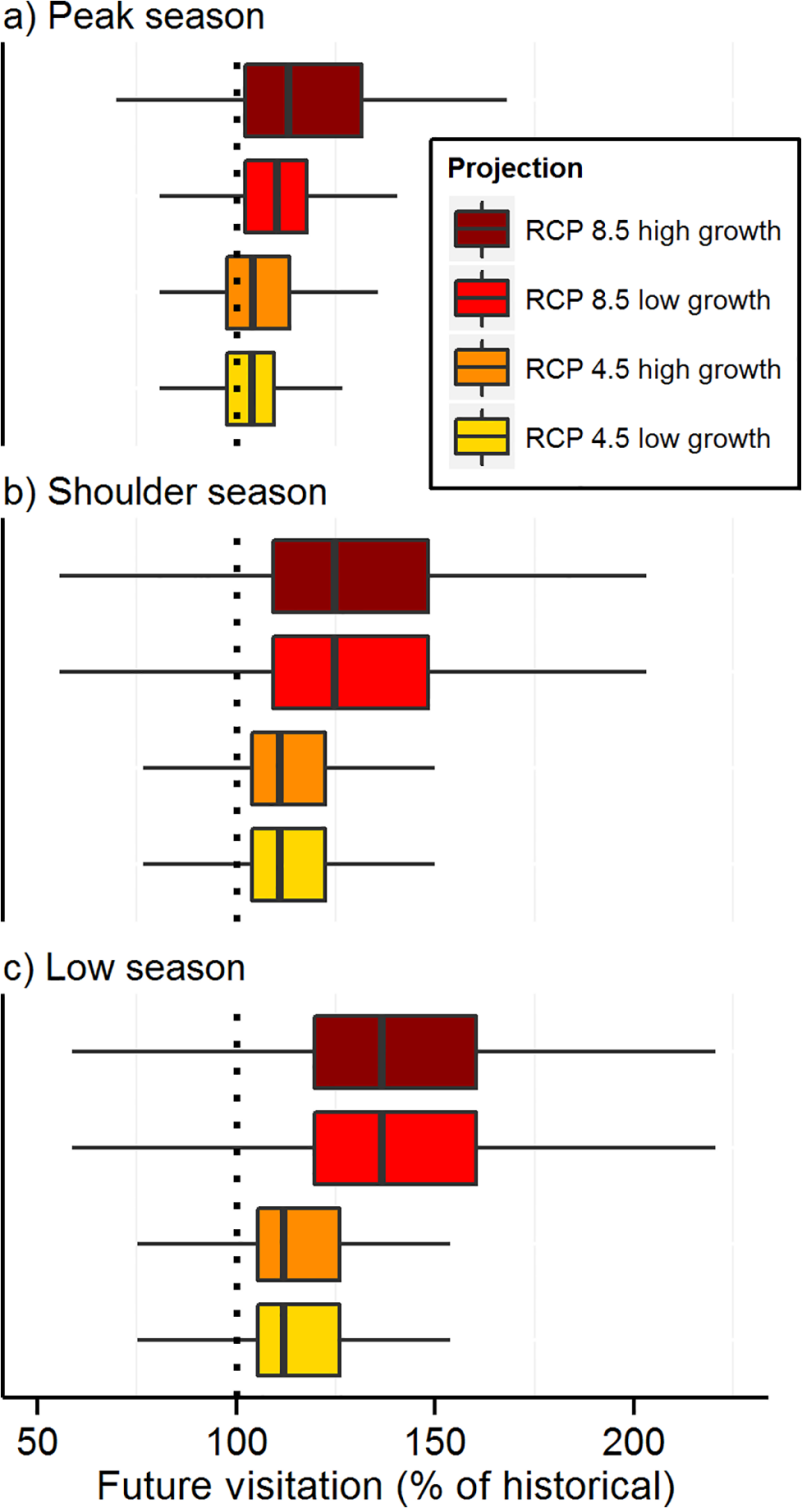
Potential changes in future visitation by climate scenario and season at U.S. national parks. Future visitation (2041–2060) at each park is expressed as a percentage of historical values (1979–2013). Future projections were limited to parks with temperature as an explanatory variable in the best-fit model and an adjusted R^2^ ≥ 0.5 (*n* = 282). Boxplots: thick vertical black line indicates median, the boxes are the interquartile range (IQR), and the whiskers extend 1.5 x IQR.

**Fig 5:**
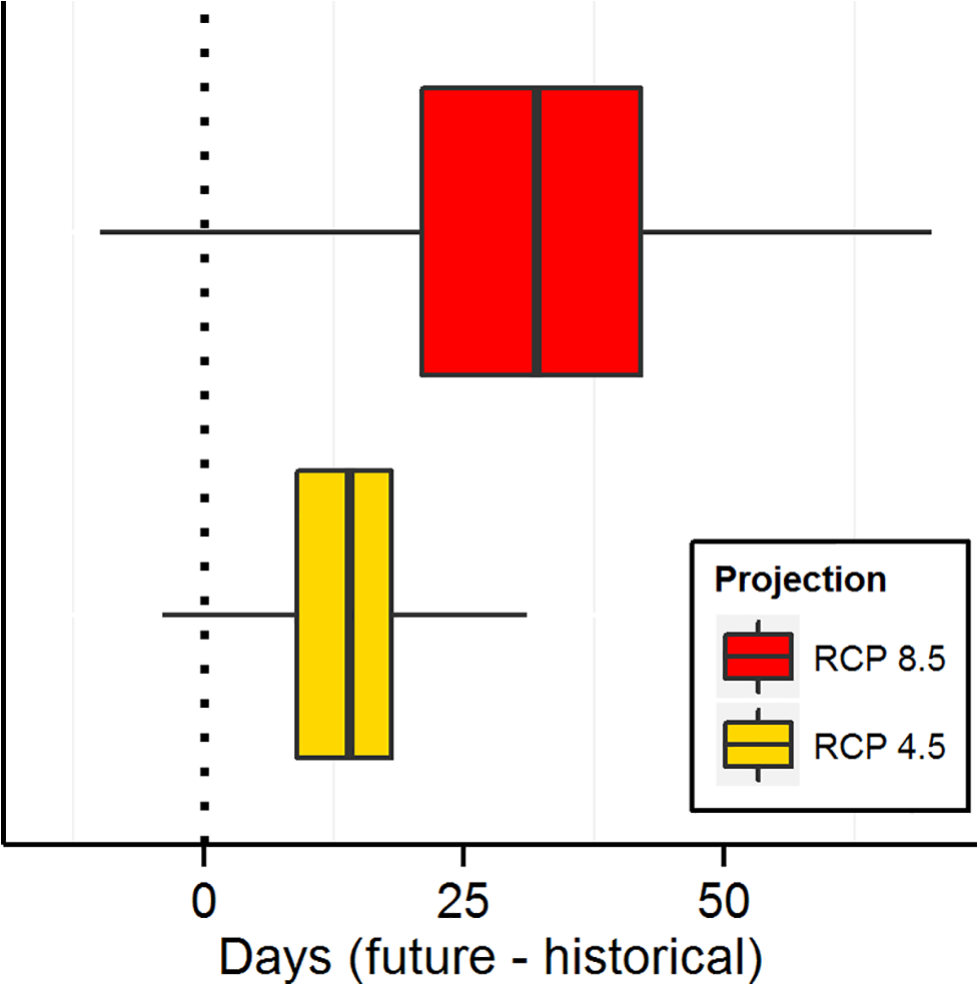
Potential future change in length (days) of the overall visitation season at parks (2041–2060 – 1979–2013). Overall visitation season length was defined as beginning on the date when 10% of historical cumulative visitation was achieved and ending on the date when 10% of historical cumulative visitation remained for the year. Projections did not vary between low- and high-maximum growth models for each RCP. Boxplots: thick vertical black line indicates median, the boxes are the interquartile range (IQR), and the whiskers extend 1.5 x IQR.

Future warming across temperature-sensitive parks is projected, on average, to increase potential total annual visits, visitation during all seasons, and the length of the visitation season (Figs 3,4 and 5, S2–S4 Figs). Future projections (2041–2060) of annual visitation at individual parks varied from < 80% to > 140% of historical values (Fig 3, S5 Fig). Most months in most parks (67–77%, RCP 4.5 low growth-RCP 8.5 high growth estimates) are projected to see warming-mediated increases in visitation, resulting in future increases (8–23%) in total annual visits across the national park system. By season, total park visits across the system are projected to increase 5–19% during the peak visitation season (three months), increase 9–24% during the shoulder seasons (two months prior to and after the peak season), and increase 13–31% during the low season (3 months). Visitation season length is projected to increase at most parks (mean increase 13–31 days). The timing of visitation (peak and low seasons) is not projected to shift temporally by more than one month at most (91–94%) temperature-sensitive parks.

## Discussion

Historical visitation is strongly related to temperature across the climatically and geographically diverse U.S. national park system, suggesting that climate change may play a large role in altering future visitation patterns in protected area networks across the globe. Higher visitation was related to warmer temperatures, except at the very warm end of the temperature spectrum (>25°C) where visitation numbers dropped off rapidly with increased temperature. These results agree with other large-scale tourism studies suggesting an inverted u-shape relationship between monthly temperature and visitation/tourism [4] and a potential poleward shift in visitation due to warming [2]. Visitation at individual parks also exhibited strong linear to polynomial relationships with monthly temperature. Future visitation at almost all parks (95%) may change based on historical visitation and temperature relationships and future climate projections. Most months in most parks are projected to climb up the visitation-temperature curve (Fig 1), resulting in potential future increases in both total annual visits and visitation season length. Although very warm months at some parks may see decreases in future visitation, this potential change represents a relatively small proportion of visitation across the national park system.

The results presented here use a single explanatory variable, monthly mean air temperature, and yet capture a large amount of the variation in visitation patterns across the system as a whole and at individual parks. The strong relationship between visitation and temperature likely reflects both direct and indirect effects (e.g., visitor comfort as a direct effect, and water for recreation from spring snowmelt as an indirect effect). Importantly, this set of parks includes the entire spectrum of NPS park types, from small historical parks (e.g., Civil War battlefields) to large wilderness parks and from rural to urban settings. Parks that do not support an historical visitation-temperature relationship include some urban parks (e.g., Klondike Goldrush in Seattle, WA), cultural parks (e.g., Moores Creek National Battlefield, NC), and tropical parks with limited monthly temperature ranges (e.g., Pu‘uhonua o Hōnaunau National Historical Park, HI).

Many factors will alter and constrain actual future visitation patterns, including population changes, economic trends, travel costs, future disposable income, international travel patterns, and the capacity of protected areas and local communities to expand services to meet changing visitor needs; further assessments of these factors are needed [29]. For example, the influence of leisure time availability, such as school vacation periods is unclear. The historical data examined here, across 340 parks and a 50°C temperature gradient, strongly suggest that temperature influences visitation choices, including reducing visitation during very warm summer school vacation months. Many parks with narrow monthly temperature gradients did not exhibit strong increases in visitation during summer months (e.g., Fig 2F), suggesting that the influence of school vacation is likely heterogeneous across the system. High visitation during spring months, when many schools in the U.S. have spring break holidays, were evident but generally only at low-latitude parks with warm spring temperatures (e.g., Fig 2E), thus supporting temperature as a major influence on where these recreation visits take place.

Beyond warming air temperatures, other factors related to climate change may affect visitation patterns. Sea-level rise may alter opportunities for some types of recreation in coastal protected areas, irrespective of temperature. Severe weather events will have strong short-term impacts on visitation, but their impacts on long-term visitation patterns are difficult to project. Phenological shifts in visitation due to phenological shifts in resources, such as the fall foliage season, were not examined here but are likely important at many protected areas. Changes to hydrologic flows, due to forces other than mean monthly temperature, may also cause visitation patterns to shift. One strength of using temperature as a predictor of future visitation is the relatively low uncertainty in projections compared with other climate and non-climate factors, such as precipitation and socio-economic conditions. Furthermore, future temperature projections used here capture plausible bookends of mid-21st century conditions.

A changing climate is likely to have cascading and complex effects on protected area visitation, management, and local economies. Recognition of the strong link between visitation and climate presents an opportunity to plan for the future and has two primary implications for protected area management and local services/economies: 1) when and where people travel will change [30, 31] and 2) the types and timing of services/facilities will need to respond to changing demands. Climate change adaptation is about adjusting to changing conditions and this includes moderating harm and exploiting beneficial opportunities [32]. Protected areas and local communities may need to do both in the future: moderate harm from too little and too much visitation (e.g., too few visitors to support local businesses or too many visitors reducing recreation enjoyment), and exploit beneficial opportunities from changing visitation patterns (e.g., increased recreation and education opportunities and visitation during shoulder seasons). Many NPS parks are already challenged with congested visitation conditions during busy weekends within the peak visitation season and this pattern may expand across additional weeks to months. Previously low-visitation periods may attract larger numbers of visitors and thus increase demands on park facilities and operations during traditionally low-staffed periods, while potentially increasing economic benefits to local communities. For parks with potentially decreasing visitation, changes in visitor services (e.g., increasing availability of potable water and air conditioning within buildings) and the timing of activities (e.g., shifts to early-morning or evening periods for outdoor activities) may be needed to adapt to such change and maintain visitation.

The NPS is about to begin its second century of preserving America’s natural and cultural heritage and providing for visitor enjoyment. The coming decades are likely to see changes in climate and changes in visitor use patterns and preferences. Protected areas and surrounding communities across the globe will need to adapt to—and may be able to capitalize on—opportunities afforded by changing visitation. Standardized historical climate data and future projections are available globally, whereas visitation data vary widely across protected areas in both availability and methodologies. Increased efforts to measure human visitation trends, the analyses provided here, and further research into short-term visitation patterns and other drivers of visitation can help protected area managers adapt to the effects of climate change and remain effective resource stewards while promoting visitor experience.

## Supporting Information

S1 Fig Strength of the relationship (adjusted R^2^ of linear model) between historical (1979–2013) monthly visitation and temperature at parks across the U.S. National Park System. Adjusted R^2^ for the best-fit model varied among parks from 0.12–0.99 (mean = 0.79, median = 0.87).
(TIF)

S2 Fig Potential future peak-season visitation (2041–2060) expressed as a percentage of historical values (1979–2013). Future projections were limited to parks with temperature as an explanatory variable in the best-fit historical model and an adjusted R^2^ ≥ 0.5 (*n* = 282). Peak season is the three contiguous months with highest historical average visitation.
(TIF)

S3 Fig Potential future shoulder-season visitation (2041–2060) expressed as a percentage of historical values (1979–2013). Future projections were limited to parks with temperature as an explanatory variable in the best-fit historical model and an adjusted R^2^ ≥ 0.5 (*n* = 282). Shoulder season is the two months before and two months after peak visitation season.
(TIF)

S4 Fig Potential future low-season visitation (2041–2060) expressed as a percentage of historical values (1979–2013). Future projections were limited to parks with temperature as an explanatory variable in the best-fit historical model and an adjusted R^2^ ≥ 0.5 (*n* = 282). Low season is the three contiguous months with lowest historical average visitation.
(TIF)

S5 Fig Potential changes in future total annual visitation by climate scenario at U.S. national parks. Future visitation (2041–2060) at each park is expressed as a percentage of historical values (1979–2013). Future projections were limited to parks with temperature as an explanatory variable in the best-fit model and an adjusted R^2^ ≥ 0.5 (*n* = 282). Boxplots: thick vertical black line indicates median, the boxes are the interquartile range (IQR), and the whiskers extend 1.5 x IQR.
(TIFF)

S1 Table The five individual climate models used in the representative concentration pathway (RCP) 4.5 W/m^2^ low climate change temperature projections for each park. These selected Coupled Model Intercomparison Project Phase 5 (CMIP5) models have the five lowest estimates of annual mean temperature out of 17 possible models for 2041–2060. Future projections were ensemble averaged across these five climate models.
(PDF)

S2 Table The five individual climate models used in the representative concentration pathway (RCP) 8.5 W/m^2^ high climate change temperature projections for each park. These selected Coupled Model Intercomparison Project Phase 5 (CMIP5) models have the five highest estimates of annual mean temperature out of 17 possible models for 2041–2060. Future projections were ensemble averaged across these five climate models.
(PDF)

S3 Table Generalized linear model output for historical (1979–2013) U.S. National Park System monthly visitation relationship to monthly mean temperature. The third-order polynomial model was the best-fit model (lowest BIC). Results shown are based on a model that includes park name as a categorical explanatory variable and a quasibinomial error distribution to account for overdispersion, and has 3737 degrees of freedom.
(PDF)

S4 Table Linear model relationship between historical monthly visitation and temperature at individual U.S. national parks. Output shown from the best-fit (lowest-BIC) model among four models of increasing complexity (a null model [‘Null’], a model including monthly temperature as the single explanatory variable [‘1^st^-order polynomial’], the second-order polynomial equation of temperature [‘2nd-order polynomial’], and the third-order polynomial equation of temperature [‘3rd-order polynomial’]).
(PDF)

## References

[1] Chape S, Harrison J, Spalding M, Lysenko I. Measuring the extent and effectiveness of protected areas as an indicator for meeting global biodiversity targets. Philos Trans R Soc Lond B Biol Sci. 2005;360: 443–455. doi: T0Y17E1UWFJPHW9D [pii]. 15814356 10.1098/rstb.2004.1592 PMC1569446

[2] Amelung B, Nicholls S, Viner D. Implications of global climate change for tourism flows and seasonality. Journal of Travel Research. 2007;45: 285–296.

[3] Scott D, Gössling S, Hall CM. International tourism and climate change. Wiley Interdisciplinary Reviews: Climate Change. 2012;3: 213–232.

[4] Rosselló-Nadal J. How to evaluate the effects of climate change on tourism. Tourism Management. 2014;42: 334–340.

[5] Stegner W, Stegner P. Marking the sparrow's fall: The making of the american west: Macmillan; 1999.

[6] National Park Service (NPS). About the National Park Service. www.nps.gov/aboutus/aboutnps.htm; 2014a.

[7] National Park Service (NPS). National Park Service Climate Change Response Strategy. Fort Collins, CO: National Park Service Climate Change Response Program; 2010.

[8] Monahan WB, Fisichelli NA. Climate exposure of US national parks in a new era of change. PLoS ONE. 2014;9(7): e101302 10.1371/journal.pone.0101302 24988483 PMC4079655

[9] Carrara PE, McGimsey RG. The late-neoglacial histories of the Agassiz and Jackson glaciers, Glacier National Park, Montana. Arct Alp Res. 1981: 183–196.

[10] Moritz C, Patton JL, Conroy CJ, Parra JL, White GC, Beissinger SR. Impact of a century of climate change on small-mammal communities in Yosemite National Park, USA. Science. 2008;322: 261–264. 10.1126/science.1163428 18845755

[11] Tingley MW, Monahan WB, Beissinger SR, Moritz C. Birds track their Grinnellian niche through a century of climate change. Proceedings of the National Academy of Sciences. 2009;106: 19637–19643. 10.1073/pnas.0901562106 PMC2780944 19805037

[12] Dolanc CR, Thorne JH, Safford HD. Widespread shifts in the demographic structure of subalpine forests in the Sierra Nevada, California, 1934 to 2007. Global Ecol Biogeogr. 2013;22: 264–276.

[13] Giersch JJ, Jordan S, Luikart F, Jones LA, Hauer FR, Muhlfeld CC. Climate-induced range contraction of a rare alpine aquatic invertebrate. Freshwater Science. 2015;: 10.1086/679490

[14] Buckley LB, Foushee MS. Footprints of climate change in US national park visitation. Int J Biometeorol. 2012;56: 1173–1177. 10.1007/s00484-011-0508-4 22109104

[15] Hadwen WL, Arthington AH, Boon PI, Taylor B, Fellows CS. Do climatic or institutional factors drive seasonal patterns of tourism visitation to protected areas across diverse climate zones in eastern australia? Tourism Geographies. 2011;13: 187–208.

[16] Albano CM, Angelo CL, Strauch RL, Thurman LL. Potential effects of warming climate on visitor use in three Alaskan national parks. Park Science. 2013;30(1): 37–44.

[17] Richardson RB, Loomis JB. Climate change and recreation benefits in an alpine national park. J Leisure Res. 2005;37: 307–320.

[18] Cullinane Thomas C, Huber C, Koontz L. 2013 national park visitor spending effects: Economic contributions to local communities, states, and the nation. 2014;Natural Resource Report NPS/NRSS/EQD/NRR-2014/824.

[19] WTTC (World Travel and Tourism Council). Travel and tourism economic impact, 2012. 2014.

[20] National Park Service (NPS). National Park Service visitor use statistics. https://irma.nps.gov/Stats/. Accessed June 2014. 2014b.

[21] Recreation Advisory Council Study Committee Number Two. A uniform method for measuring and reporting recreation use on the public lands and waters of the united states. 1965: 56.

[22] Harris I, Jones P, Osborn T, Lister D. Updated high‐resolution grids of monthly climatic observations–the CRU TS3. 10 dataset. Int J Climatol. 2014;34: 623–642.

[23] Hijmans RJ, Cameron SE, Parra JL, Jones PG, Jarvis A. Very high resolution interpolated climate surfaces for global land areas. Int J Climatol. 2005;25: 1965–1978.

[24] Mieczkowski Z. The tourism climatic index: A method of evaluating world climates for tourism. The Canadian Geographer/Le Géographe canadien. 1985;29: 220–233.

[25] Endler C, Oehler K, Matzarakis A. Vertical gradient of climate change and climate tourism conditions in the black forest. Int J Biometeorol. 2010;54: 45–61. 10.1007/s00484-009-0251-2 19705164

[26] Scott D, Jones B, Konopek J. Implications of climate and environmental change for nature-based tourism in the canadian rocky mountains: A case study of Waterton Lakes National Park. Tourism Management. 2007;28: 570–579.

[27] Schwarz G. Estimating the dimension of a model. The Annals of Statistics. 1978;6: 461–464.

[28] R Core Team. R: A language and environment for statistical computing. R Foundation for Statistical Computing. 2014;R package version 1.1–7, http://CRAN.R-project.org/package=lme4.

[29] Gössling S, Scott D, Hall CM, Ceron J, Dubois G. Consumer behaviour and demand response of tourists to climate change. Ann Tourism Res. 2012;39: 36–58.

[30] Bigano A, Hamilton JM, Maddison DJ, Tol RS. Predicting tourism flows under climate change. Clim Change. 2006;79: 175–180.

[31] Scott D, Lemieux C. Weather and climate information for tourism. Procedia Environmental Sciences. 2010;1: 146–183.

[32] Intergovernmental Panel on Climate Change (IPCC). Summary for policymakers In: Field CB, Barros VR, Mach KJ, Mastrandrea M, Bilir TE, Chatterjee M, et al, editors. Climate Change 2014: Impacts, Adaptation, and Vulnerability. Part A: Global and Sectoral Aspects. Contribution of Working Group II to the Fifth Assessment Report of the Intergovernmental Panel on Climate Change. Cambridge, United Kingdom and New York, NY, USA: Cambridge University Press; 2014.

