# Supplementary figures and images for: Protected Area Tourism in a Changing Climate: Will Visitation at US National Parks Warm Up or Overheat?
Source: PLoS One. 2015 Jun 17;10(6):e0128226. doi: 10.1371/journal.pone.0128226 (PMC4470629; doi:10.1371/journal.pone.0128226)

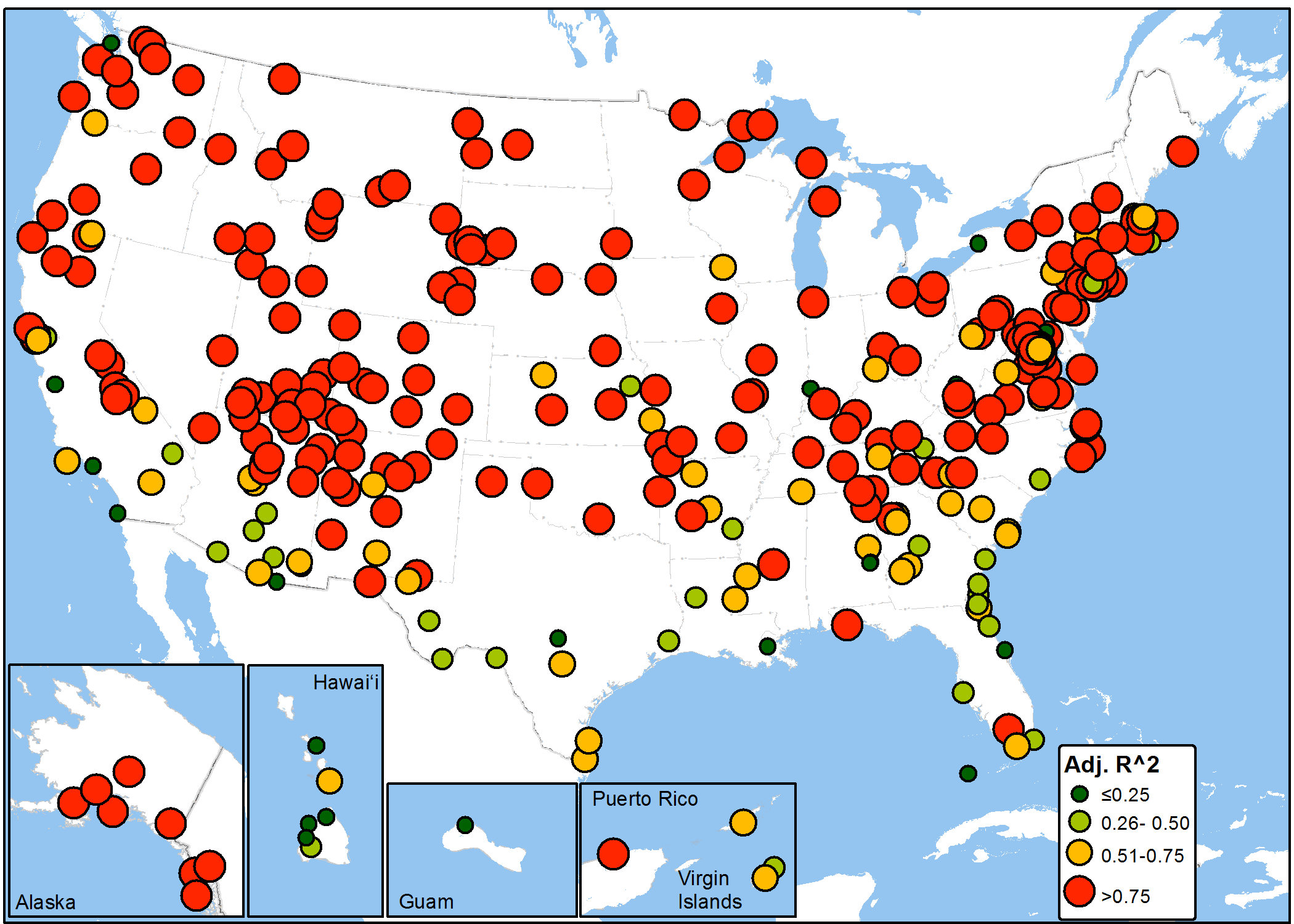

Supplement: S1 Fig — Adjusted R2 for the best-fit model varied among parks from 0.12–0.99 (mean = 0.79, median = 0.87). (TIF) [file pone.0128226.s001.tif]

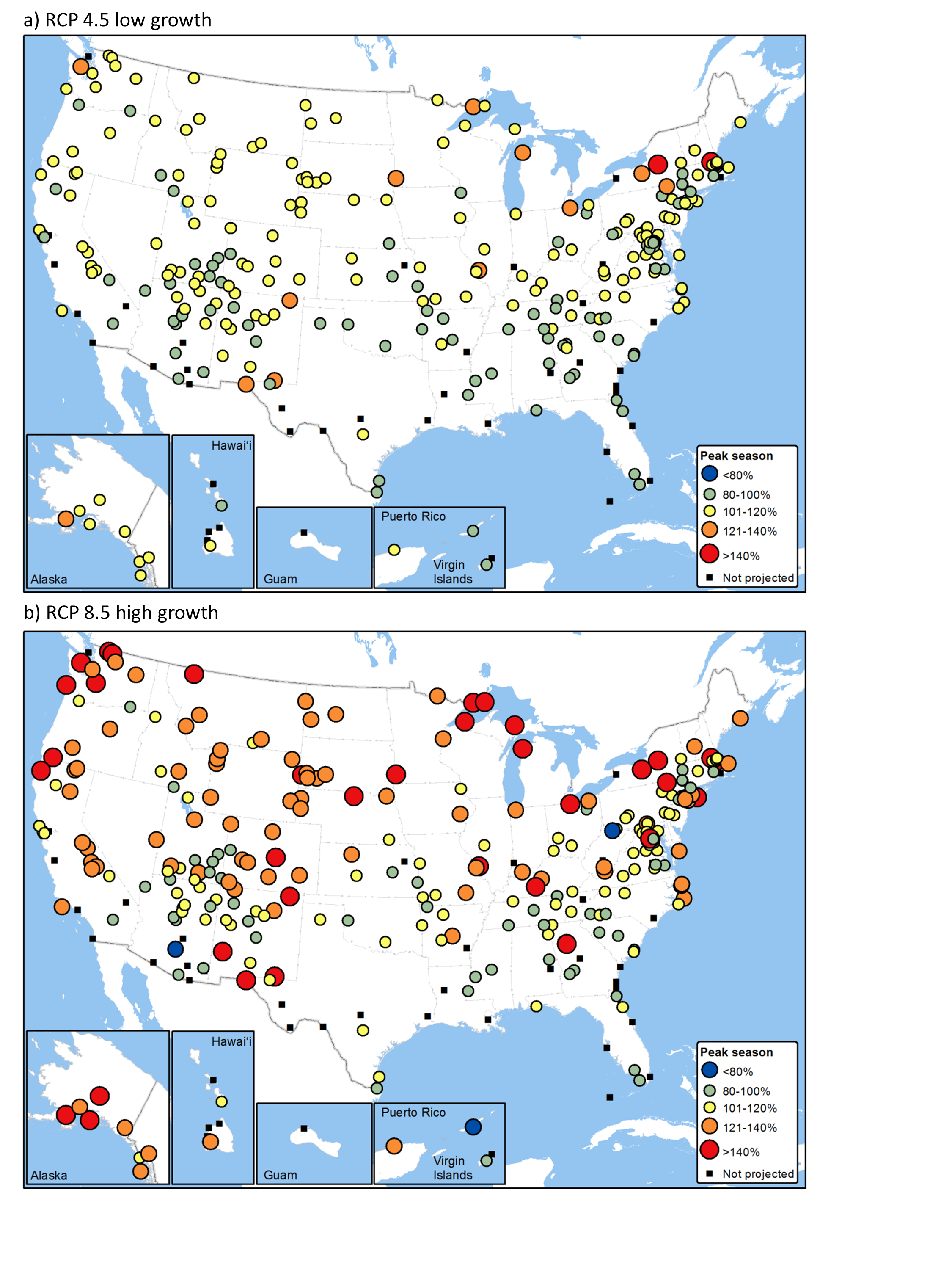

Supplement: S2 Fig — Future projections were limited to parks with temperature as an explanatory variable in the best-fit historical model and an adjusted R2 ≥ 0.5 (n = 282). Peak season is the three contiguous months with highest historical average visitation. (TIF) [file pone.0128226.s002.tif]

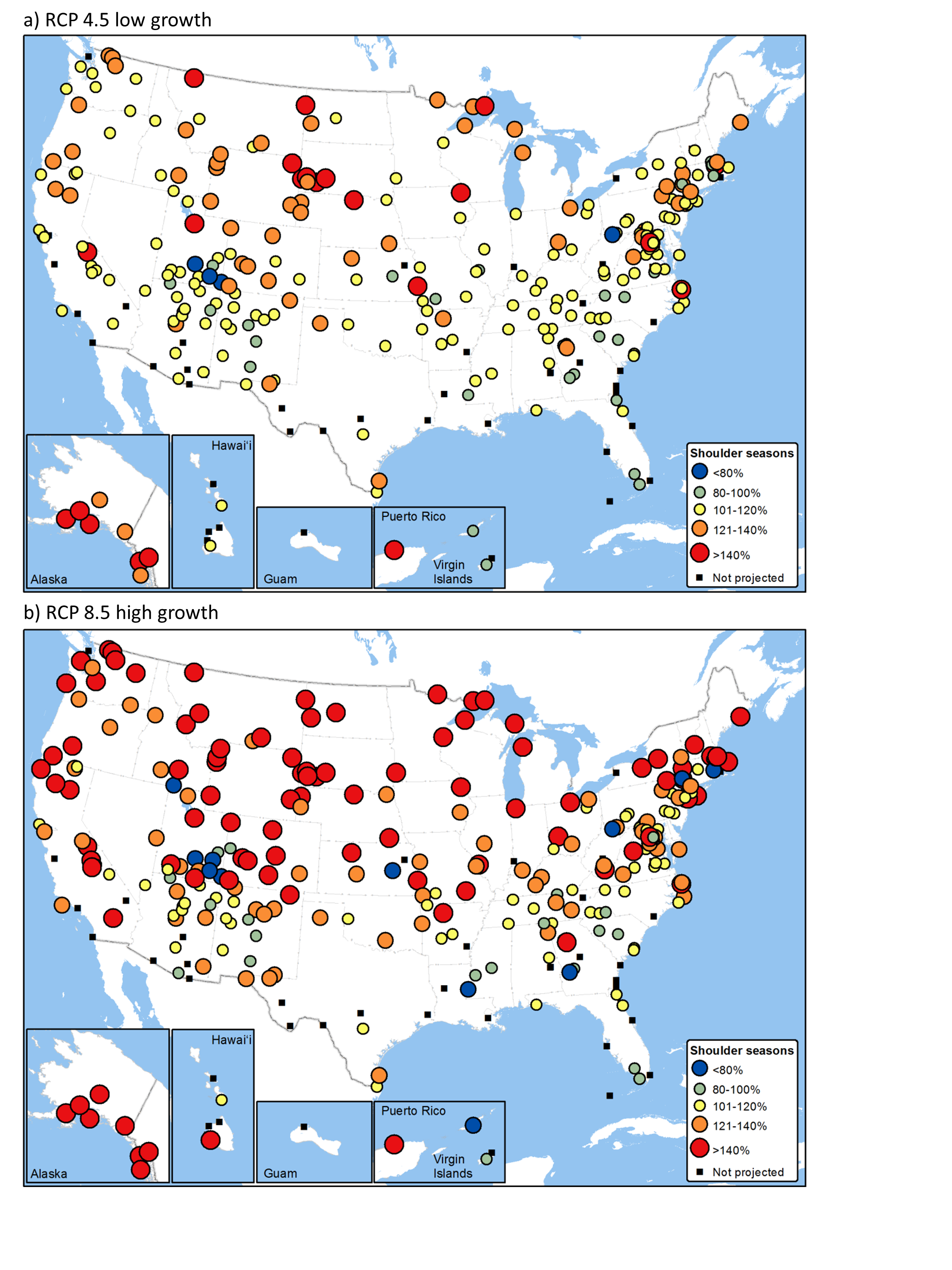

Supplement: S3 Fig — Future projections were limited to parks with temperature as an explanatory variable in the best-fit historical model and an adjusted R2 ≥ 0.5 (n = 282). Shoulder season is the two months before and two months after peak visitation season. (TIF) [file pone.0128226.s003.tif]

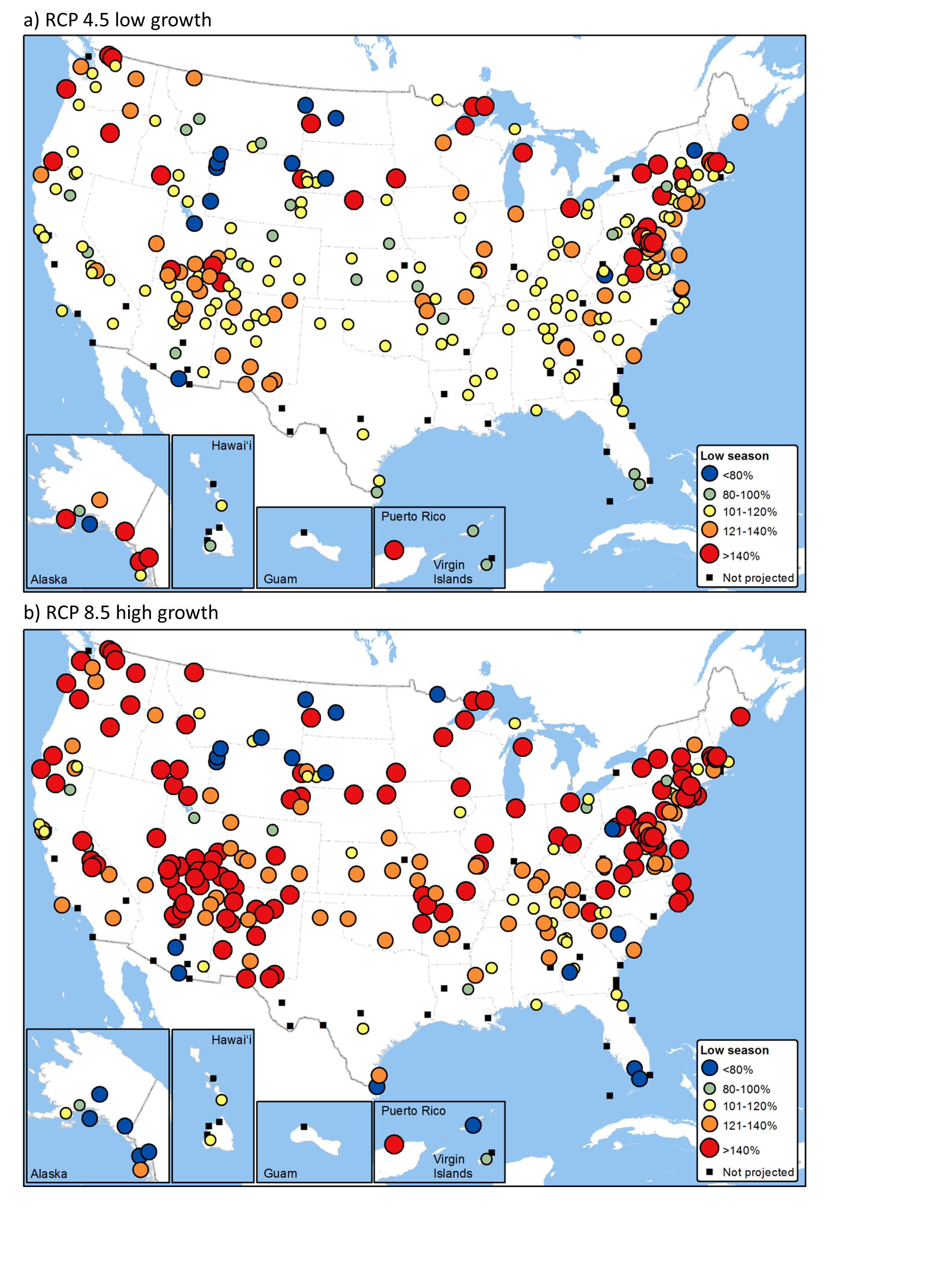

Supplement: S4 Fig — Future projections were limited to parks with temperature as an explanatory variable in the best-fit historical model and an adjusted R2 ≥ 0.5 (n = 282). Low season is the three contiguous months with lowest historical average visitation. (TIF) [file pone.0128226.s004.tif]

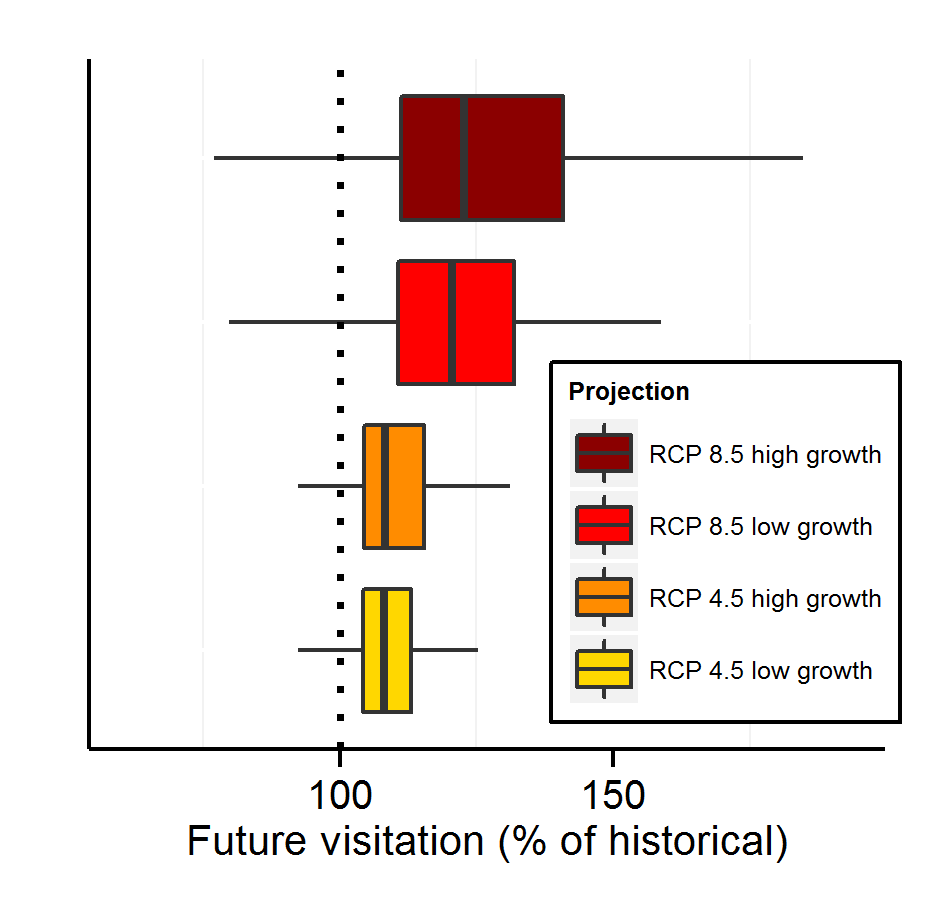

Supplement: S5 Fig — Future visitation (2041–2060) at each park is expressed as a percentage of historical values (1979–2013). Future projections were limited to parks with temperature as an explanatory variable in the best-fit model and an adjusted R2 ≥ 0.5 (n = 282). Boxplots: thick vertical black line indicates median, the boxes are the interquartile range (IQR), and the whiskers extend 1.5 x IQR. (TIFF) [file pone.0128226.s005.tiff]
