# Supplementary material for: Protected Area Tourism in a Changing Climate: Will Visitation at US National Parks Warm Up or Overheat?
Source: PLoS One. 2015 Jun 17;10(6):e0128226. doi: 10.1371/journal.pone.0128226 (PMC4470629; doi:10.1371/journal.pone.0128226)
Supplement: S1 Table — These selected Coupled Model Intercomparison Project Phase 5 (CMIP5) models have the five lowest estimates of annual mean temperature out of 17 possible models for 2041–2060. Future projections were ensemble averaged across these five climate models. (PDF) [file pone.0128226.s006.pdf]

S1 Table. The five individual climate models used in the representative concentration pathway (RCP) 4.5 W/m^2 low climate change temperature projections for each park. These selected Coupled Model Intercomparison Project Phase 5 (CMIP5) models have the five lowest estimates of annual mean temperature out of 17 possible models for 2041-2060. Future projections were ensemble averaged across these five climate models.

Park	Model 1	Model 2	Model 3	Model 4	Model 5
Abraham Lincoln Birthplace National Historic Site	MRI-CGCM3	INMCM4	CNRM-CM5	MPI-ESM-LR	GISS-E2-R
Acadia National Park	INMCM4	GISS-E2-R	MRI-CGCM3	NorESM1-M	CCSM4
Adams National Historical Park	INMCM4	GISS-E2-R	MRI-CGCM3	NorESM1-M	BCC-CSM1-1
Agate Fossil Beds National Monument	INMCM4	MRI-CGCM3	GISS-E2-R	NorESM1-M	CCSM4
Allegheny Portage Railroad National Historic Site	INMCM4	MRI-CGCM3	NorESM1-M	CNRM-CM5	GISS-E2-R
Andersonville National Historical Site	INMCM4	MRI-CGCM3	CNRM-CM5	MPI-ESM-LR	NorESM1-M
Antietam National Battlefield	INMCM4	MRI-CGCM3	NorESM1-M	CNRM-CM5	MPI-ESM-LR
Appomattox Court House National Historical Park	INMCM4	MRI-CGCM3	CNRM-CM5	MPI-ESM-LR	GISS-E2-R
Apostle Islands National Lakeshore	INMCM4	MRI-CGCM3	GISS-E2-R	NorESM1-M	BCC-CSM1-1
Arches National Park	INMCM4	MRI-CGCM3	GISS-E2-R	BCC-CSM1-1	MPI-ESM-LR
Arlington House / The Robert E. Lee National Memorial	INMCM4	MRI-CGCM3	CNRM-CM5	NorESM1-M	GISS-E2-R
Assateague Island National Seashore	INMCM4	MRI-CGCM3	CNRM-CM5	CCSM4	MPI-ESM-LR
Aztec Ruins National Monument	INMCM4	MRI-CGCM3	GISS-E2-R	BCC-CSM1-1	CCSM4
Badlands National Park	MRI-CGCM3	INMCM4	GISS-E2-R	NorESM1-M	MPI-ESM-LR
Bandelier National Monument	INMCM4	MRI-CGCM3	CCSM4	GISS-E2-R	BCC-CSM1-1
Bent's Old Fort National Historic Site	INMCM4	MRI-CGCM3	GISS-E2-R	NorESM1-M	CCSM4
Bighorn Canyon National Recreation Area	INMCM4	MRI-CGCM3	GISS-E2-R	MPI-ESM-LR	CNRM-CM5

Park	Model 1	Model 2	Model 3	Model 4	Model 5
Big Cypress National Preserve	INMCM4	MRI-CGCM3	CNRM-CM5	NorESM1-M	BCC-CSM1-1
Big Hole National Battlefield	MRI-CGCM3	INMCM4	GISS-E2-R	CNRM-CM5	MPI-ESM-LR
Big South Fork National River and Recreation Area	MRI-CGCM3	INMCM4	CNRM-CM5	MPI-ESM-LR	NorESM1-M
Black Canyon of the Gunnison National Park	MRI-CGCM3	INMCM4	GISS-E2-R	BCC-CSM1-1	CNRM-CM5
Blue Ridge Parkway	INMCM4	MRI-CGCM3	CNRM-CM5	MPI-ESM-LR	NorESM1-M
Bluestone National Scenic River	MRI-CGCM3	INMCM4	CNRM-CM5	MPI-ESM-LR	GISS-E2-R
Boston African American National Historic Site	INMCM4	GISS-E2-R	MRI-CGCM3	NorESM1-M	BCC-CSM1-1
Boston National Historical Park	INMCM4	GISS-E2-R	MRI-CGCM3	NorESM1-M	BCC-CSM1-1
Booker T. Washington National Monument	INMCM4	MRI-CGCM3	CNRM-CM5	MPI-ESM-LR	GISS-E2-R
Bryce Canyon National Park	INMCM4	MRI-CGCM3	GISS-E2-R	MPI-ESM-LR	BCC-CSM1-1
Buffalo National River	MRI-CGCM3	INMCM4	MIROC-ESM	CNRM-CM5	GISS-E2-R
Canyon de Chelly National Monument	INMCM4	MRI-CGCM3	GISS-E2-R	BCC-CSM1-1	CCSM4
Castle Clinton National Monument	INMCM4	MRI-CGCM3	NorESM1-M	GISS-E2-R	CNRM-CM5
Cape Cod National Seashore	INMCM4	GISS-E2-R	CCSM4	NorESM1-M	MRI-CGCM3
Casa Grande Ruins National Monument	INMCM4	MRI-CGCM3	GISS-E2-R	BCC-CSM1-1	CNRM-CM5
Cape Hatteras National Seashore	INMCM4	MRI-CGCM3	CNRM-CM5	MPI-ESM-LR	NorESM1-M
Cape Lookout National Seashore	INMCM4	MRI-CGCM3	CNRM-CM5	MPI-ESM-LR	ACCESS1-0
Canyonlands National Park	INMCM4	MRI-CGCM3	GISS-E2-R	BCC-CSM1-1	MPI-ESM-LR
Capitol Reef National Park	INMCM4	MRI-CGCM3	GISS-E2-R	MPI-ESM-LR	BCC-CSM1-1
Carl Sandburg Home National Historic Site	INMCM4	MRI-CGCM3	CNRM-CM5	MPI-ESM-LR	NorESM1-M
Catoctin Mountain Park	INMCM4	MRI-CGCM3	NorESM1-M	CNRM-CM5	GISS-E2-R

Park	Model 1	Model 2	Model 3	Model 4	Model 5
Carlsbad Caverns National Park	INMCM4	MRI-CGCM3	BCC-CSM1-1	CCSM4	GISS-E2-R
Capulin Volcano National Monument	INMCM4	MRI-CGCM3	CCSM4	GISS-E2-R	MPI-ESM-LR
Cedar Breaks National Monument	INMCM4	MRI-CGCM3	GISS-E2-R	MPI-ESM-LR	BCC-CSM1-1
Chamizal National Memorial	INMCM4	MRI-CGCM3	BCC-CSM1-1	CCSM4	GISS-E2-R
Chattahoochee River National Recreation Area	INMCM4	MRI-CGCM3	CNRM-CM5	MPI-ESM-LR	NorESM1-M
Chickamauga and Chattanooga National Military Park	INMCM4	MRI-CGCM3	CNRM-CM5	MPI-ESM-LR	GISS-E2-R
Chaco Culture National Historical Park	INMCM4	MRI-CGCM3	GISS-E2-R	BCC-CSM1-1	CCSM4
Chickasaw National Recreation Area	INMCM4	MRI-CGCM3	GISS-E2-R	CCSM4	CNRM-CM5
Channel Islands National Park	INMCM4	MRI-CGCM3	GISS-E2-R	HadGEM2-CC	CCSM4
Chesapeake and Ohio Canal National Historic Park	INMCM4	MRI-CGCM3	NorESM1-M	CNRM-CM5	MPI-ESM-LR
Charles Pinckney National Historic Site	INMCM4	MRI-CGCM3	CNRM-CM5	MPI-ESM-LR	BCC-CSM1-1
Christiansted National Historic Site	INMCM4	BCC-CSM1-1	CNRM-CM5	MRI-CGCM3	CCSM4
Little Rock Central High School National Historic Site	INMCM4	MRI-CGCM3	MIROC-ESM	CNRM-CM5	MPI-ESM-LR
City of Rocks National Reserve	MRI-CGCM3	INMCM4	GISS-E2-R	MPI-ESM-LR	BCC-CSM1-1
Colorado National Monument	INMCM4	MRI-CGCM3	GISS-E2-R	BCC-CSM1-1	CNRM-CM5
Colonial National Historical Park	INMCM4	MRI-CGCM3	CNRM-CM5	NorESM1-M	CCSM4
Congaree National Park	INMCM4	MRI-CGCM3	CNRM-CM5	MIROC-ESM-CHEM	MPI-ESM-LR
Cowpens National Battlefield	INMCM4	MRI-CGCM3	CNRM-CM5	MPI-ESM-LR	GISS-E2-R
Crater Lake National Park	INMCM4	MRI-CGCM3	GISS-E2-R	MIROC5	CNRM-CM5

Park	Model 1	Model 2	Model 3	Model 4	Model 5
Craters of the Moon National Monument	MRI-CGCM3	INMCM4	GISS-E2-R	MPI-ESM-LR	CNRM-CM5
Cumberland Gap National Historical Park	MRI-CGCM3	INMCM4	CNRM-CM5	MPI-ESM-LR	NorESM1-M
Curecanti National Recreation Area	MRI-CGCM3	INMCM4	GISS-E2-R	MPI-ESM-LR	CNRM-CM5
Cuyahoga Valley National Park	INMCM4	MRI-CGCM3	NorESM1-M	CCSM4	MPI-ESM-LR
Dayton Aviation Heritage National Historical Park	INMCM4	MRI-CGCM3	GISS-E2-R	MPI-ESM-LR	NorESM1-M
Denali National Park & Preserve	MRI-CGCM3	INMCM4	GISS-E2-R	MIROC5	BCC-CSM1-1
Devils Postpile National Monument	INMCM4	MRI-CGCM3	GISS-E2-R	BCC-CSM1-1	CCSM4
Devils Tower National Monument	MRI-CGCM3	INMCM4	GISS-E2-R	MPI-ESM-LR	NorESM1-M
Death Valley National Park	INMCM4	MRI-CGCM3	GISS-E2-R	CNRM-CM5	MPI-ESM-LR
Delaware Water Gap National Recreation Area	INMCM4	MRI-CGCM3	NorESM1-M	CNRM-CM5	GISS-E2-R
Dinosaur National Monument	MRI-CGCM3	INMCM4	GISS-E2-R	CNRM-CM5	BCC-CSM1-1
Edison National Historic Site	INMCM4	MRI-CGCM3	NorESM1-M	GISS-E2-R	CNRM-CM5
Effigy Mounds National Monument	MRI-CGCM3	INMCM4	CNRM-CM5	GISS-E2-R	BCC-CSM1-1
Eisenhower National Historic Site	INMCM4	MRI-CGCM3	NorESM1-M	CNRM-CM5	GISS-E2-R
El Malpais National Monument	INMCM4	MRI-CGCM3	CCSM4	GISS-E2-R	BCC-CSM1-1
El Morro National Monument	INMCM4	MRI-CGCM3	BCC-CSM1-1	CCSM4	GISS-E2-R
Eleanor Roosevelt National Historic Site	INMCM4	MRI-CGCM3	GISS-E2-R	NorESM1-M	CNRM-CM5
Everglades National Park	INMCM4	MRI-CGCM3	CNRM-CM5	NorESM1-M	BCC-CSM1-1
Federal Hall National Memorial	INMCM4	MRI-CGCM3	NorESM1-M	GISS-E2-R	CNRM-CM5
Fire Island National Seashore	INMCM4	GISS-E2-R	NorESM1-M	MRI-CGCM3	BCC-CSM1-1
Florissant Fossil Beds National Monument	INMCM4	MRI-CGCM3	GISS-E2-R	CCSM4	MPI-ESM-LR

Park	Model 1	Model 2	Model 3	Model 4	Model 5
Fort Bowie National Historic Site	INMCM4	MRI-CGCM3	BCC-CSM1-1	GISS-E2-R	CCSM4
Fossil Butte National Monument	MRI-CGCM3	INMCM4	GISS-E2-R	CNRM-CM5	BCC-CSM1-1
Fort Caroline National Memorial	INMCM4	MRI-CGCM3	CNRM-CM5	MPI-ESM-LR	NorESM1-M
Fort Donelson National Battlefield	MRI-CGCM3	INMCM4	CNRM-CM5	MPI-ESM-LR	GISS-E2-R
Fort Laramie National Historic Site	INMCM4	MRI-CGCM3	GISS-E2-R	MPI-ESM-LR	NorESM1-M
Fort Larned National Historic Site	INMCM4	MRI-CGCM3	GISS-E2-R	CNRM-CM5	NorESM1-M
Fort Matanzas National Monument	INMCM4	MRI-CGCM3	CNRM-CM5	NorESM1-M	BCC-CSM1-1
Fort McHenry National Monument and Historic Shrine	INMCM4	MRI-CGCM3	CNRM-CM5	NorESM1-M	GISS-E2-R
Fort Necessity National Battlefield	MRI-CGCM3	INMCM4	NorESM1-M	CNRM-CM5	GISS-E2-R
Fort Point National Historic Site	MRI-CGCM3	INMCM4	GISS-E2-R	HadGEM2-CC	CCSM4
Fort Raleigh National Historical Site	INMCM4	MRI-CGCM3	CNRM-CM5	MPI-ESM-LR	NorESM1-M
Fort Scott National Historic Site	MRI-CGCM3	INMCM4	CNRM-CM5	GISS-E2-R	CCSM4
Fort Smith National Historic Site	INMCM4	MRI-CGCM3	MIROC-ESM	CNRM-CM5	GISS-E2-R
Fort Stanwix National Monument	INMCM4	MRI-CGCM3	CNRM-CM5	GISS-E2-R	NorESM1-M
Fort Sumter National Monument	INMCM4	MRI-CGCM3	CNRM-CM5	MPI-ESM-LR	BCC-CSM1-1
Fort Union National Monument	INMCM4	MRI-CGCM3	CCSM4	GISS-E2-R	BCC-CSM1-1
Fort Union Trading Post National Historic Site	INMCM4	MRI-CGCM3	GISS-E2-R	BCC-CSM1-1	CNRM-CM5
Fort Vancouver National Historic Site	INMCM4	GISS-E2-R	MRI-CGCM3	CNRM-CM5	MIROC5
Fort Washington Park	INMCM4	MRI-CGCM3	CNRM-CM5	NorESM1-M	GISS-E2-R
Friendship Hill National Historic Site	MRI-CGCM3	INMCM4	NorESM1-M	CNRM-CM5	GISS-E2-R

Park	Model 1	Model 2	Model 3	Model 4	Model 5
Fredericksburg and Spotsylvania National Military Park	INMCM4	MRI-CGCM3	CNRM-CM5	NorESM1-M	MPI-ESM-LR
Gateway National Recreation Area	INMCM4	MRI-CGCM3	NorESM1-M	GISS-E2-R	CNRM-CM5
General Grant National Memorial	INMCM4	MRI-CGCM3	GISS-E2-R	NorESM1-M	CNRM-CM5
Gettysburg National Military Park	INMCM4	MRI-CGCM3	NorESM1-M	CNRM-CM5	GISS-E2-R
George Washington Birthplace National Monument	INMCM4	MRI-CGCM3	CNRM-CM5	NorESM1-M	CCSM4
Gila Cliff Dwellings National Monument	INMCM4	MRI-CGCM3	BCC-CSM1-1	CCSM4	GISS-E2-R
Glacier National Park	INMCM4	MRI-CGCM3	GISS-E2-R	CNRM-CM5	MPI-ESM-LR
Glacier Bay National Park & Preserve	GISS-E2-R	INMCM4	MRI-CGCM3	MIROC5	HadGEM2-CC
Glen Canyon National Recreation Area	INMCM4	MRI-CGCM3	GISS-E2-R	MPI-ESM-LR	BCC-CSM1-1
Golden Gate National Recreation Area	INMCM4	MRI-CGCM3	GISS-E2-R	HadGEM2-CC	CCSM4
Governors Island National Monument	INMCM4	MRI-CGCM3	NorESM1-M	GISS-E2-R	CNRM-CM5
Golden Spike National Historic Site	MRI-CGCM3	INMCM4	GISS-E2-R	BCC-CSM1-1	MPI-ESM-LR
Great Basin National Park	INMCM4	MRI-CGCM3	GISS-E2-R	MPI-ESM-LR	BCC-CSM1-1
Grand Canyon National Park	INMCM4	MRI-CGCM3	GISS-E2-R	MPI-ESM-LR	CCSM4
Greenbelt Park	INMCM4	MRI-CGCM3	CNRM-CM5	NorESM1-M	GISS-E2-R
Grant-Kohrs Ranch National Historic Site	INMCM4	MRI-CGCM3	GISS-E2-R	CNRM-CM5	MPI-ESM-LR
Grand Portage National Monument	INMCM4	MRI-CGCM3	GISS-E2-R	NorESM1-M	BCC-CSM1-1
Great Sand Dunes National Park	MRI-CGCM3	INMCM4	GISS-E2-R	CCSM4	MPI-ESM-LR
Great Smoky Mountains National Park	INMCM4	MRI-CGCM3	CNRM-CM5	MPI-ESM-LR	NorESM1-M
Grand Teton National Park	MRI-CGCM3	INMCM4	GISS-E2-R	CNRM-CM5	MPI-ESM-LR

Park	Model 1	Model 2	Model 3	Model 4	Model 5
Guilford Courthouse National Military Park	INMCM4	MRI-CGCM3	CNRM-CM5	MPI-ESM-LR	NorESM1-M
Gulf Islands National Seashore	INMCM4	MRI-CGCM3	CNRM-CM5	NorESM1-M	MPI-ESM-LR
Guadalupe Mountains National Park	INMCM4	MRI-CGCM3	BCC-CSM1-1	GISS-E2-R	CCSM4
George Washington Carver National Monument	MRI-CGCM3	INMCM4	CNRM-CM5	GISS-E2-R	MIROC-ESM
George Washington Memorial Parkway	INMCM4	MRI-CGCM3	CNRM-CM5	NorESM1-M	GISS-E2-R
Harpers Ferry National Historical Park	INMCM4	MRI-CGCM3	CNRM-CM5	MPI-ESM-LR	NorESM1-M
Hagerman Fossil Beds National Monument	INMCM4	MRI-CGCM3	GISS-E2-R	MPI-ESM-LR	BCC-CSM1-1
Hamilton Grange National Memorial	INMCM4	MRI-CGCM3	GISS-E2-R	NorESM1-M	CNRM-CM5
Haleakala National Park	INMCM4	MRI-CGCM3	GISS-E2-R	ACCESS1-0	CNRM-CM5
Hawai'i Volcanoes National Park	INMCM4	MRI-CGCM3	GISS-E2-R	MIROC5	CNRM-CM5
Herbert Hoover National Historic Site	MRI-CGCM3	INMCM4	CNRM-CM5	GISS-E2-R	MPI-ESM-LR
Horseshoe Bend National Military Park	INMCM4	MRI-CGCM3	CNRM-CM5	MPI-ESM-LR	GISS-E2-R
Hopewell Culture National Historical Park	INMCM4	MRI-CGCM3	NorESM1-M	MPI-ESM-LR	CNRM-CM5
Home of Franklin D. Roosevelt National Historic Site	INMCM4	MRI-CGCM3	GISS-E2-R	NorESM1-M	CNRM-CM5
Hopewell Furnace National Historic Site	INMCM4	MRI-CGCM3	NorESM1-M	CNRM-CM5	GISS-E2-R
Homestead National Monument	MRI-CGCM3	INMCM4	GISS-E2-R	CNRM-CM5	CCSM4
Hot Springs National Park	INMCM4	MRI-CGCM3	MIROC-ESM	CNRM-CM5	MPI-ESM-LR
Hovenweep National Monument	INMCM4	MRI-CGCM3	GISS-E2-R	BCC-CSM1-1	CNRM-CM5
Harry S. Truman National Historic Site	MRI-CGCM3	INMCM4	CNRM-CM5	GISS-E2-R	CCSM4
Hubbell Trading Post National Historic Site	INMCM4	MRI-CGCM3	GISS-E2-R	BCC-CSM1-1	CCSM4

Park	Model 1	Model 2	Model 3	Model 4	Model 5
Independence National Historical Park	INMCM4	MRI-CGCM3	NorESM1-M	GISS-E2-R	CNRM-CM5
Indiana Dunes National Lakeshore	INMCM4	MRI-CGCM3	CNRM-CM5	NorESM1-M	GISS-E2-R
Isle Royale National Park	INMCM4	MRI-CGCM3	GISS-E2-R	NorESM1-M	CCSM4
James A Garfield National Historic Site	INMCM4	MRI-CGCM3	NorESM1-M	CCSM4	CNRM-CM5
Jewel Cave National Monument	INMCM4	MRI-CGCM3	GISS-E2-R	MPI-ESM-LR	NorESM1-M
Jefferson National Expansion Memorial National Memorial	MRI-CGCM3	INMCM4	CNRM-CM5	GISS-E2-R	CCSM4
Jimmy Carter National Historic Site	INMCM4	MRI-CGCM3	CNRM-CM5	MPI-ESM-LR	NorESM1-M
John Day Fossil Beds National Monument	INMCM4	MRI-CGCM3	GISS-E2-R	MPI-ESM-LR	MIROC5
John D. Rockefeller, Jr. Memorial Parkway	MRI-CGCM3	INMCM4	GISS-E2-R	CNRM-CM5	MPI-ESM-LR
John Fitzgerald Kennedy National Historic Site	INMCM4	GISS-E2-R	MRI-CGCM3	NorESM1-M	BCC-CSM1-1
Johnstown Flood National Memorial	INMCM4	MRI-CGCM3	NorESM1-M	CNRM-CM5	GISS-E2-R
Joshua Tree National Park	INMCM4	MRI-CGCM3	GISS-E2-R	MPI-ESM-LR	CNRM-CM5
Katmai National Park & Preserve	MIROC5	MRI-CGCM3	INMCM4	GISS-E2-R	MIROC-ESM-CHEM
Kenai Fjords National Park	MRI-CGCM3	MIROC5	GISS-E2-R	INMCM4	CNRM-CM5
Kennesaw Mountain National Battlefield Park	INMCM4	MRI-CGCM3	CNRM-CM5	MPI-ESM-LR	GISS-E2-R
Kings Canyon National Park	INMCM4	MRI-CGCM3	GISS-E2-R	BCC-CSM1-1	MPI-ESM-LR
Kings Mountain National Military Park	INMCM4	MRI-CGCM3	CNRM-CM5	MPI-ESM-LR	GISS-E2-R
Klondike Gold Rush AK National Historical Park	MRI-CGCM3	GISS-E2-R	INMCM4	CNRM-CM5	MIROC5
Klondike Gold Rush WA National Historical Park	INMCM4	MRI-CGCM3	GISS-E2-R	CNRM-CM5	MIROC5
Knife River Indian Villages National Historic Site	MRI-CGCM3	INMCM4	GISS-E2-R	BCC-CSM1-1	CNRM-CM5

Park	Model 1	Model 2	Model 3	Model 4	Model 5
Korean War Veterans Memorial National Memorial	INMCM4	MRI-CGCM3	CNRM-CM5	NorESM1-M	GISS-E2-R
Lava Beds National Monument	INMCM4	MRI-CGCM3	GISS-E2-R	MIROC5	BCC-CSM1-1
Lake Chelan National Recreation Area	INMCM4	MRI-CGCM3	GISS-E2-R	MIROC5	CNRM-CM5
Lake Clark National Park & Preserve	MRI-CGCM3	GISS-E2-R	MIROC5	INMCM4	BCC-CSM1-1
Lake Mead National Recreation Area	INMCM4	MRI-CGCM3	GISS-E2-R	MPI-ESM-LR	CNRM-CM5
Lake Meredith National Recreation Area	INMCM4	MRI-CGCM3	GISS-E2-R	CCSM4	CNRM-CM5
Lake Roosevelt National Recreation Area	INMCM4	GISS-E2-R	MRI-CGCM3	MIROC5	CNRM-CM5
Lassen Volcanic National Park	INMCM4	MRI-CGCM3	GISS-E2-R	MIROC5	CCSM4
Lewis and Clark National Historical Trail	GISS-E2-R	INMCM4	MRI-CGCM3	CNRM-CM5	MIROC5
Little Bighorn Battlefield National Monument	INMCM4	MRI-CGCM3	GISS-E2-R	MPI-ESM-LR	CCSM4
Lincoln Boyhood National Memorial	INMCM4	MRI-CGCM3	CNRM-CM5	GISS-E2-R	MPI-ESM-LR
Lincoln Home National Historic Site	INMCM4	MRI-CGCM3	CNRM-CM5	GISS-E2-R	NorESM1-M
Lincoln Memorial National Memorial	INMCM4	MRI-CGCM3	CNRM-CM5	NorESM1-M	GISS-E2-R
Little River Canyon National Preserve	INMCM4	MRI-CGCM3	CNRM-CM5	MPI-ESM-LR	NorESM1-M
Longfellow National Historic Site	INMCM4	GISS-E2-R	MRI-CGCM3	NorESM1-M	BCC-CSM1-1
Lowell National Historical Park	INMCM4	GISS-E2-R	MRI-CGCM3	NorESM1-M	BCC-CSM1-1
Lyndon Baines Johnson Memorial Grove on the Potomac National Memorial	INMCM4	MRI-CGCM3	CNRM-CM5	NorESM1-M	GISS-E2-R
Mammoth Cave National Park	INMCM4	MRI-CGCM3	CNRM-CM5	MPI-ESM-LR	GISS-E2-R
Martin Luther King, Jr. National Historic Site	INMCM4	MRI-CGCM3	CNRM-CM5	MPI-ESM-LR	GISS-E2-R

Park	Model 1	Model 2	Model 3	Model 4	Model 5
Manassas National Battlefield Park	INMCM4	MRI-CGCM3	CNRM-CM5	NorESM1-M	GISS-E2-R
Manzanar National Historic Site	INMCM4	MRI-CGCM3	GISS-E2-R	BCC-CSM1-1	CCSM4
Martin Van Buren National Historic Site	INMCM4	MRI-CGCM3	GISS-E2-R	NorESM1-M	CNRM-CM5
Mesa Verde National Park	INMCM4	MRI-CGCM3	GISS-E2-R	BCC-CSM1-1	CNRM-CM5
Minute Man National Historical Park	INMCM4	GISS-E2-R	MRI-CGCM3	NorESM1-M	BCC-CSM1-1
Minuteman Missile National Historic Site	MRI-CGCM3	INMCM4	GISS-E2-R	NorESM1-M	MPI-ESM-LR
Missouri National Recreation River	MRI-CGCM3	INMCM4	GISS-E2-R	CNRM-CM5	BCC-CSM1-1
Montezuma Castle National Monument	INMCM4	MRI-CGCM3	GISS-E2-R	BCC-CSM1-1	CCSM4
Monocacy National Battlefield	INMCM4	MRI-CGCM3	CNRM-CM5	GISS-E2-R	MPI-ESM-LR
Mount Rainier National Park	INMCM4	MRI-CGCM3	GISS-E2-R	MIROC5	CNRM-CM5
Morristown National Historical Park	INMCM4	MRI-CGCM3	NorESM1-M	GISS-E2-R	CNRM-CM5
Mount Rushmore National Memorial	INMCM4	MRI-CGCM3	GISS-E2-R	NorESM1-M	CCSM4
Muir Woods National Monument	INMCM4	MRI-CGCM3	GISS-E2-R	HadGEM2-CC	CCSM4
Natural Bridges National Monument	INMCM4	MRI-CGCM3	GISS-E2-R	BCC-CSM1-1	MPI-ESM-LR
National Capital combined Park	INMCM4	MRI-CGCM3	CNRM-CM5	NorESM1-M	GISS-E2-R
Natchez National Historical Park	INMCM4	MRI-CGCM3	CNRM-CM5	MPI-ESM-LR	GISS-E2-R
Natchez Trace Parkway and National Scenic Trail	INMCM4	MRI-CGCM3	CNRM-CM5	MPI-ESM-LR	GISS-E2-R
Navajo National Monument	INMCM4	MRI-CGCM3	GISS-E2-R	BCC-CSM1-1	MPI-ESM-LR
Nez Perce National Historical Park	INMCM4	MRI-CGCM3	GISS-E2-R	MPI-ESM-LR	CNRM-CM5
New River Gorge National River	MRI-CGCM3	INMCM4	CNRM-CM5	MPI-ESM-LR	GISS-E2-R
Nicodemus National Historic Site	INMCM4	MRI-CGCM3	GISS-E2-R	CNRM-CM5	NorESM1-M

Park	Model 1	Model 2	Model 3	Model 4	Model 5
Niobrara National Scenic River	MRI-CGCM3	INMCM4	GISS-E2-R	CNRM-CM5	NorESM1-M
Ninety Six National Historic Site	INMCM4	MRI-CGCM3	CNRM-CM5	MPI-ESM-LR	NorESM1-M
North Cascades National Park	INMCM4	MRI-CGCM3	GISS-E2-R	MIROC5	CNRM-CM5
Obed Wild and Scenic River	INMCM4	MRI-CGCM3	CNRM-CM5	MPI-ESM-LR	NorESM1-M
Olympic National Park	INMCM4	MRI-CGCM3	GISS-E2-R	CNRM-CM5	MIROC5
Oregon Caves National Monument	MRI-CGCM3	INMCM4	GISS-E2-R	CNRM-CM5	MIROC5
Ozark National Scenic Riverway	MRI-CGCM3	INMCM4	CNRM-CM5	GISS-E2-R	MPI-ESM-LR
Palo Alto Battlefield National Historic Site	INMCM4	MRI-CGCM3	BCC-CSM1-1	GISS-E2-R	CNRM-CM5
Pennsylvania Avenue National Historic Site National Historic Site	INMCM4	MRI-CGCM3	CNRM-CM5	NorESM1-M	GISS-E2-R
Padre Island National Seashore	INMCM4	MRI-CGCM3	GISS-E2-R	BCC-CSM1-1	CNRM-CM5
Pecos National Historic Park	INMCM4	MRI-CGCM3	CCSM4	GISS-E2-R	BCC-CSM1-1
Petrified Forest National Park	INMCM4	MRI-CGCM3	BCC-CSM1-1	CCSM4	GISS-E2-R
Pea Ridge National Military Park	MRI-CGCM3	INMCM4	CNRM-CM5	MIROC-ESM	GISS-E2-R
Petersburg National Battlefield	INMCM4	MRI-CGCM3	CNRM-CM5	MPI-ESM-LR	NorESM1-M
Petroglyph National Monument	INMCM4	MRI-CGCM3	CCSM4	GISS-E2-R	BCC-CSM1-1
Perry's Victory and International Peace Memorial	INMCM4	MRI-CGCM3	NorESM1-M	CNRM-CM5	GISS-E2-R
Pipestone National Monument	MRI-CGCM3	INMCM4	GISS-E2-R	CNRM-CM5	BCC-CSM1-1
Pictured Rocks National Lakeshore	INMCM4	GISS-E2-R	NorESM1-M	MRI-CGCM3	CCSM4
Piscataway Park	INMCM4	MRI-CGCM3	CNRM-CM5	NorESM1-M	GISS-E2-R
Pipe Spring National Monument	INMCM4	MRI-CGCM3	GISS-E2-R	CCSM4	MPI-ESM-LR

Park	Model 1	Model 2	Model 3	Model 4	Model 5
Point Reyes National Seashore	INMCM4	MRI-CGCM3	GISS-E2-R	HadGEM2-CC	CCSM4
Prince William Forest Park	INMCM4	MRI-CGCM3	CNRM-CM5	NorESM1-M	GISS-E2-R
Rainbow Bridge National Monument	INMCM4	MRI-CGCM3	GISS-E2-R	BCC-CSM1-1	MPI-ESM-LR
Redwood National Park	MRI-CGCM3	INMCM4	GISS-E2-R	MIROC5	CNRM-CM5
Richmond National Battlefield Park	INMCM4	MRI-CGCM3	CNRM-CM5	NorESM1-M	MPI-ESM-LR
Rock Creek Park Park	INMCM4	MRI-CGCM3	CNRM-CM5	NorESM1-M	GISS-E2-R
Ross Lake National Recreation Area	INMCM4	MRI-CGCM3	GISS-E2-R	CNRM-CM5	MIROC5
Rocky Mountain National Park	INMCM4	MRI-CGCM3	GISS-E2-R	MPI-ESM-LR	CCSM4
Roger Williams National Memorial	INMCM4	GISS-E2-R	MRI-CGCM3	NorESM1-M	BCC-CSM1-1
Russell Cave National Monument	INMCM4	MRI-CGCM3	CNRM-CM5	MPI-ESM-LR	NorESM1-M
San Antonio Missions National Historic Park	INMCM4	MRI-CGCM3	GISS-E2-R	CCSM4	ACCESS1-0
Saint Croix National Scenic River	INMCM4	MRI-CGCM3	CNRM-CM5	GISS-E2-R	BCC-CSM1-1
San Francisco Maritime National Historical Park	MRI-CGCM3	INMCM4	GISS-E2-R	HadGEM2-CC	CCSM4
Saint-Gaudens National Historic Site	INMCM4	MRI-CGCM3	GISS-E2-R	CNRM-CM5	NorESM1-M
Sagamore Hill National Historic Site	INMCM4	MRI-CGCM3	GISS-E2-R	NorESM1-M	CNRM-CM5
Saugus Iron Works National Historic Site	INMCM4	GISS-E2-R	MRI-CGCM3	NorESM1-M	BCC-CSM1-1
San Juan National Historical Site	INMCM4	BCC-CSM1-1	CNRM-CM5	MRI-CGCM3	CCSM4
Salem Maritime National Historic Site	INMCM4	GISS-E2-R	MRI-CGCM3	NorESM1-M	CCSM4
Salinas Pueblo Missions National Monument	INMCM4	MRI-CGCM3	CCSM4	BCC-CSM1-1	GISS-E2-R
Saratoga National Historical Park	INMCM4	MRI-CGCM3	GISS-E2-R	NorESM1-M	CNRM-CM5
Scotts Bluff National Monument	INMCM4	MRI-CGCM3	GISS-E2-R	NorESM1-M	CCSM4

Park	Model 1	Model 2	Model 3	Model 4	Model 5
Sequoia National Park	INMCM4	MRI-CGCM3	GISS-E2-R	CNRM-CM5	BCC-CSM1-1
Shenandoah National Park	INMCM4	MRI-CGCM3	CNRM-CM5	NorESM1-M	GISS-E2-R
Shiloh National Battlefield	INMCM4	MRI-CGCM3	CNRM-CM5	MPI-ESM-LR	GISS-E2-R
Sitka National Historical Park	INMCM4	MRI-CGCM3	GISS-E2-R	MIROC5	HadGEM2-CC
Sleeping Bear Dunes National Lakeshore	INMCM4	GISS-E2-R	NorESM1-M	MRI-CGCM3	BCC-CSM1-1
Springfield Armory National Historic Site	INMCM4	MRI-CGCM3	GISS-E2-R	NorESM1-M	CNRM-CM5
Steamtown National Historic Site	INMCM4	MRI-CGCM3	NorESM1-M	CNRM-CM5	GISS-E2-R
Statue Of Liberty National Monument	INMCM4	MRI-CGCM3	NorESM1-M	GISS-E2-R	CNRM-CM5
Stones River National Battlefield	INMCM4	MRI-CGCM3	CNRM-CM5	MPI-ESM-LR	GISS-E2-R
Sunset Crater Volcano National Monument	INMCM4	MRI-CGCM3	GISS-E2-R	BCC-CSM1-1	CCSM4
Tallgrass Prairie National Preserve	MRI-CGCM3	INMCM4	GISS-E2-R	CNRM-CM5	CCSM4
Theodore Roosevelt Island National Memorial	INMCM4	MRI-CGCM3	CNRM-CM5	NorESM1-M	GISS-E2-R
Theodore Roosevelt National Park	MRI-CGCM3	INMCM4	GISS-E2-R	CNRM-CM5	BCC-CSM1-1
Timpanogos Cave National Monument	MRI-CGCM3	INMCM4	GISS-E2-R	MPI-ESM-LR	BCC-CSM1-1
Tumacacori National Historical Park	INMCM4	MRI-CGCM3	GISS-E2-R	BCC-CSM1-1	CCSM4
Tuzigoot National Monument	INMCM4	MRI-CGCM3	GISS-E2-R	BCC-CSM1-1	CCSM4
Ulysses S. Grant National Historic Site	MRI-CGCM3	INMCM4	CNRM-CM5	GISS-E2-R	CCSM4
Upper Delaware Scenic and Recreational River	INMCM4	MRI-CGCM3	NorESM1-M	GISS-E2-R	CNRM-CM5
Valley Forge National Historical Park	INMCM4	MRI-CGCM3	GISS-E2-R	NorESM1-M	CNRM-CM5
World War II Valor in the Pacific National Monument National Monument	INMCM4	MRI-CGCM3	GISS-E2-R	MIROC5	CNRM-CM5

Park	Model 1	Model 2	Model 3	Model 4	Model 5
Vanderbilt Mansion National Historic Site	INMCM4	MRI-CGCM3	GISS-E2-R	NorESM1-M	CNRM-CM5
Vicksburg National Military Park	INMCM4	MRI-CGCM3	CNRM-CM5	MPI-ESM-LR	GISS-E2-R
Virgin Islands National Park	INMCM4	BCC-CSM1-1	CNRM-CM5	MRI-CGCM3	CCSM4
Vietnam Veterans Memorial National Memorial	INMCM4	MRI-CGCM3	CNRM-CM5	NorESM1-M	GISS-E2-R
Voyageurs National Park	INMCM4	MRI-CGCM3	GISS-E2-R	CNRM-CM5	BCC-CSM1-1
Washita Battlefield National Historic Site	INMCM4	MRI-CGCM3	GISS-E2-R	CNRM-CM5	CCSM4
Walnut Canyon National Monument	INMCM4	MRI-CGCM3	GISS-E2-R	BCC-CSM1-1	CCSM4
Weir Farm National Historic Site	INMCM4	MRI-CGCM3	GISS-E2-R	NorESM1-M	BCC-CSM1-1
Whiskeytown National Recreation Area	MRI-CGCM3	INMCM4	GISS-E2-R	MIROC5	CNRM-CM5
Whitman Mission National Historic Site	INMCM4	GISS-E2-R	MRI-CGCM3	CNRM-CM5	MPI-ESM-LR
White Sands National Monument	INMCM4	MRI-CGCM3	BCC-CSM1-1	CCSM4	GISS-E2-R
Wind Cave National Park	MRI-CGCM3	INMCM4	GISS-E2-R	NorESM1-M	MPI-ESM-LR
Wilson's Creek National Battlefield	MRI-CGCM3	INMCM4	CNRM-CM5	GISS-E2-R	MIROC-ESM
William Howard Taft National Historic Site	INMCM4	MRI-CGCM3	CNRM-CM5	GISS-E2-R	MPI-ESM-LR
Women's Rights National Historical Park	INMCM4	MRI-CGCM3	CNRM-CM5	NorESM1-M	CCSM4
Wolf Trap National Park for the Performing Arts	INMCM4	MRI-CGCM3	CNRM-CM5	NorESM1-M	GISS-E2-R
Wright Brothers National Memorial	INMCM4	MRI-CGCM3	CNRM-CM5	GISS-E2-R	MPI-ESM-LR
Wrangell-St. Elias National Park & Preserve	MRI-CGCM3	INMCM4	GISS-E2-R	CNRM-CM5	MIROC5
Wupatki National Monument	INMCM4	MRI-CGCM3	GISS-E2-R	MPI-ESM-LR	BCC-CSM1-1
World War II Memorial National Memorial	INMCM4	MRI-CGCM3	CNRM-CM5	NorESM1-M	GISS-E2-R

Park	Model 1	Model 2	Model 3	Model 4	Model 5
Yellowstone National Park	MRI-CGCM3	INMCM4	GISS-E2-R	CNRM-CM5	MPI-ESM-LR
Yosemite National Park	INMCM4	MRI-CGCM3	GISS-E2-R	CCSM4	BCC-CSM1-1
Zion National Park	INMCM4	MRI-CGCM3	GISS-E2-R	MPI-ESM-LR	BCC-CSM1-1
