# Supplementary material for: Protected Area Tourism in a Changing Climate: Will Visitation at US National Parks Warm Up or Overheat?
Source: PLoS One. 2015 Jun 17;10(6):e0128226. doi: 10.1371/journal.pone.0128226 (PMC4470629; doi:10.1371/journal.pone.0128226)
Supplement: S2 Table — These selected Coupled Model Intercomparison Project Phase 5 (CMIP5) models have the five highest estimates of annual mean temperature out of 17 possible models for 2041–2060. Future projections were ensemble averaged across these five climate models. (PDF) [file pone.0128226.s007.pdf]

S2 Table. **The five individual climate models used in the representative concentration pathway (RCP) 8.5 W/m^2 high climate change temperature projections for each park.** These selected Coupled Model Intercomparison Project Phase 5 (CMIP5) models have the five highest estimates of annual mean temperature out of 17 possible models for 2041-2060. Future projections were ensemble averaged across these five climate models.

Park	Model 1	Model 2	Model 3	Model 4	Model 5
Abraham Lincoln Birthplace National Historic Site	HadGEM2-ES	ACCESS1-0	MIROC-ESM	HadGEM2-AO	MIROC-ESM-CHEM
Acadia National Park	MIROC-ESM-CHEM	MIROC-ESM	GFDL-CM3	HadGEM2-ES	ACCESS1-0
Adams National Historical Park	MIROC-ESM-CHEM	MIROC-ESM	GFDL-CM3	MIROC5	HadGEM2-ES
Agate Fossil Beds National Monument	HadGEM2-AO	ACCESS1-0	MIROC-ESM	MIROC-ESM-CHEM	HadGEM2-ES
Allegheny Portage Railroad National Historic Site	GFDL-CM3	MIROC-ESM	MIROC-ESM-CHEM	HadGEM2-ES	ACCESS1-0
Andersonville National Historical Site	MIROC-ESM-CHEM	HadGEM2-ES	HadGEM2-CC	ACCESS1-0	GFDL-CM3
Antietam National Battlefield	MIROC-ESM	GFDL-CM3	HadGEM2-ES	MIROC-ESM-CHEM	ACCESS1-0
Appomattox Court House National Historical Park	MIROC-ESM	HadGEM2-ES	GFDL-CM3	HadGEM2-CC	ACCESS1-0
Apostle Islands National Lakeshore	MIROC-ESM	MIROC-ESM-CHEM	ACCESS1-0	GFDL-CM3	HadGEM2-ES
Arches National Park	MIROC-ESM-CHEM	MIROC-ESM	GFDL-CM3	HadGEM2-AO	MIROC5
Arlington House / The Robert E. Lee National Memorial	MIROC-ESM	HadGEM2-ES	GFDL-CM3	MIROC-ESM-CHEM	ACCESS1-0
Assateague Island National Seashore	MIROC-ESM-CHEM	MIROC-ESM	GFDL-CM3	IPSL-CM5A-LR	MIROC5

Park	Model 1	Model 2	Model 3	Model 4	Model 5
Aztec Ruins National Monument	MIROC-ESM-CHEM	GFDL-CM3	MIROC-ESM	IPSL-CM5A-LR	MIROC5
Badlands National Park	HadGEM2-AO	ACCESS1-0	MIROC-ESM	MIROC-ESM-CHEM	HadGEM2-ES
Bandelier National Monument	MIROC-ESM-CHEM	MIROC-ESM	IPSL-CM5A-LR	GFDL-CM3	HadGEM2-ES
Bent's Old Fort National Historic Site	MIROC-ESM-CHEM	IPSL-CM5A-LR	HadGEM2-ES	HadGEM2-AO	ACCESS1-0
Bighorn Canyon National Recreation Area	MIROC-ESM-CHEM	MIROC-ESM	HadGEM2-AO	ACCESS1-0	HadGEM2-ES
Big Cypress National Preserve	MIROC-ESM-CHEM	GFDL-CM3	MIROC-ESM	HadGEM2-ES	IPSL-CM5A-LR
Big Hole National Battlefield	MIROC-ESM-CHEM	MIROC-ESM	IPSL-CM5A-LR	HadGEM2-AO	GFDL-CM3
Big South Fork National River and Recreation Area	HadGEM2-ES	GFDL-CM3	ACCESS1-0	HadGEM2-CC	HadGEM2-AO
Black Canyon of the Gunnison National Park	GFDL-CM3	MIROC-ESM-CHEM	IPSL-CM5A-LR	MIROC-ESM	MIROC5
Blue Ridge Parkway	GFDL-CM3	HadGEM2-ES	HadGEM2-CC	ACCESS1-0	MIROC-ESM
Bluestone National Scenic River	GFDL-CM3	HadGEM2-ES	ACCESS1-0	MIROC-ESM	HadGEM2-CC
Boston African American National Historic Site	MIROC-ESM-CHEM	MIROC-ESM	GFDL-CM3	MIROC5	HadGEM2-ES
Boston National Historical Park	MIROC-ESM-CHEM	MIROC-ESM	GFDL-CM3	MIROC5	HadGEM2-ES
Booker T. Washington National Monument	GFDL-CM3	HadGEM2-ES	MIROC-ESM	HadGEM2-CC	ACCESS1-0
Bryce Canyon National Park	MIROC-ESM-CHEM	MIROC-ESM	HadGEM2-AO	IPSL-CM5A-LR	HadGEM2-ES

Park	Model 1	Model 2	Model 3	Model 4	Model 5
Buffalo National River	MIROC-ESM-CHEM	ACCESS1-0	MIROC-ESM	HadGEM2-ES	HadGEM2-AO
Canyon de Chelly National Monument	MIROC-ESM-CHEM	MIROC-ESM	GFDL-CM3	IPSL-CM5A-LR	HadGEM2-AO
Castle Clinton National Monument	MIROC-ESM	MIROC-ESM-CHEM	GFDL-CM3	MIROC5	HadGEM2-ES
Cape Cod National Seashore	MIROC-ESM-CHEM	MIROC-ESM	GFDL-CM3	HadGEM2-ES	MIROC5
Casa Grande Ruins National Monument	MIROC-ESM-CHEM	IPSL-CM5A-LR	MIROC-ESM	HadGEM2-ES	GFDL-CM3
Cape Hatteras National Seashore	GFDL-CM3	MIROC-ESM-CHEM	MIROC-ESM	IPSL-CM5A-LR	HadGEM2-ES
Cape Lookout National Seashore	GFDL-CM3	MIROC-ESM-CHEM	MIROC-ESM	IPSL-CM5A-LR	HadGEM2-ES
Canyonlands National Park	MIROC-ESM-CHEM	MIROC-ESM	GFDL-CM3	HadGEM2-AO	IPSL-CM5A-LR
Capitol Reef National Park	MIROC-ESM-CHEM	MIROC-ESM	HadGEM2-AO	GFDL-CM3	IPSL-CM5A-LR
Carl Sandburg Home National Historic Site	HadGEM2-ES	GFDL-CM3	HadGEM2-CC	MIROC-ESM	ACCESS1-0
Catoctin Mountain Park	MIROC-ESM	GFDL-CM3	HadGEM2-ES	MIROC-ESM-CHEM	ACCESS1-0
Carlsbad Caverns National Park	IPSL-CM5A-LR	MIROC-ESM-CHEM	HadGEM2-ES	GFDL-CM3	HadGEM2-CC
Capulin Volcano National Monument	MIROC-ESM-CHEM	IPSL-CM5A-LR	HadGEM2-ES	ACCESS1-0	MIROC-ESM
Cedar Breaks National Monument	MIROC-ESM-CHEM	MIROC-ESM	HadGEM2-AO	IPSL-CM5A-LR	HadGEM2-ES

Park	Model 1	Model 2	Model 3	Model 4	Model 5
Chamizal National Memorial	IPSL-CM5A-LR	MIROC-ESM-CHEM	HadGEM2-ES	HadGEM2-CC	GFDL-CM3
Chattahoochee River National Recreation Area	HadGEM2-ES	HadGEM2-CC	HadGEM2-AO	MIROC-ESM	GFDL-CM3
Chickamauga and Chattanooga National Military Park	HadGEM2-ES	MIROC-ESM	HadGEM2-CC	GFDL-CM3	ACCESS1-0
Chaco Culture National Historical Park	MIROC-ESM-CHEM	GFDL-CM3	MIROC-ESM	IPSL-CM5A-LR	MIROC5
Chickasaw National Recreation Area	MIROC-ESM-CHEM	IPSL-CM5A-LR	MIROC5	ACCESS1-0	HadGEM2-AO
Channel Islands National Park	MIROC-ESM-CHEM	IPSL-CM5A-LR	MIROC-ESM	HadGEM2-ES	GFDL-CM3
Chesapeake and Ohio Canal National Historic Park	MIROC-ESM	GFDL-CM3	HadGEM2-ES	ACCESS1-0	MIROC-ESM-CHEM
Charles Pinckney National Historic Site	MIROC-ESM	GFDL-CM3	MIROC-ESM-CHEM	IPSL-CM5A-LR	HadGEM2-ES
Christiansted National Historic Site	IPSL-CM5A-LR	GFDL-CM3	MIROC-ESM	MIROC-ESM-CHEM	HadGEM2-ES
Little Rock Central High School National Historic Site	MIROC-ESM-CHEM	MIROC-ESM	HadGEM2-ES	HadGEM2-AO	ACCESS1-0
City of Rocks National Reserve	MIROC-ESM-CHEM	MIROC-ESM	HadGEM2-AO	ACCESS1-0	IPSL-CM5A-LR
Colorado National Monument	MIROC-ESM-CHEM	GFDL-CM3	MIROC-ESM	ACCESS1-0	MIROC5
Colonial National Historical Park	MIROC-ESM	MIROC-ESM-CHEM	GFDL-CM3	ACCESS1-0	IPSL-CM5A-LR
Congaree National Park	HadGEM2-ES	HadGEM2-CC	MIROC-ESM	GFDL-CM3	ACCESS1-0
Cowpens National Battlefield	HadGEM2-ES	HadGEM2-CC	GFDL-CM3	ACCESS1-0	MIROC-ESM

Park	Model 1	Model 2	Model 3	Model 4	Model 5
Crater Lake National Park	HadGEM2-AO	IPSL-CM5A-LR	HadGEM2-ES	MIROC-ESM-CHEM	MIROC-ESM
Craters of the Moon National Monument	MIROC-ESM-CHEM	MIROC-ESM	HadGEM2-AO	GFDL-CM3	ACCESS1-0
Cumberland Gap National Historical Park	HadGEM2-ES	GFDL-CM3	ACCESS1-0	HadGEM2-CC	MIROC-ESM
Curecanti National Recreation Area	GFDL-CM3	MIROC-ESM-CHEM	IPSL-CM5A-LR	MIROC5	ACCESS1-0
Cuyahoga Valley National Park	MIROC-ESM	HadGEM2-ES	MIROC-ESM-CHEM	ACCESS1-0	HadGEM2-CC
Dayton Aviation Heritage National Historical Park	MIROC-ESM	HadGEM2-ES	ACCESS1-0	MIROC-ESM-CHEM	HadGEM2-AO
Denali National Park & Preserve	GFDL-CM3	HadGEM2-ES	MIROC-ESM-CHEM	ACCESS1-0	HadGEM2-CC
Devils Postpile National Monument	MIROC-ESM	MIROC-ESM-CHEM	IPSL-CM5A-LR	HadGEM2-AO	GFDL-CM3
Devils Tower National Monument	HadGEM2-AO	ACCESS1-0	MIROC-ESM-CHEM	HadGEM2-ES	MIROC-ESM
Death Valley National Park	MIROC-ESM	MIROC-ESM-CHEM	IPSL-CM5A-LR	HadGEM2-AO	GFDL-CM3
Delaware Water Gap National Recreation Area	MIROC-ESM	MIROC-ESM-CHEM	GFDL-CM3	HadGEM2-ES	ACCESS1-0
Dinosaur National Monument	MIROC-ESM-CHEM	MIROC-ESM	GFDL-CM3	HadGEM2-AO	ACCESS1-0
Edison National Historic Site	MIROC-ESM	MIROC-ESM-CHEM	GFDL-CM3	MIROC5	HadGEM2-ES
Effigy Mounds National Monument	MIROC-ESM	MIROC-ESM-CHEM	ACCESS1-0	HadGEM2-ES	HadGEM2-AO

Park	Model 1	Model 2	Model 3	Model 4	Model 5
Eisenhower National Historic Site	MIROC-ESM	GFDL-CM3	HadGEM2-ES	MIROC5	MIROC-ESM-CHEM
El Malpais National Monument	MIROC-ESM-CHEM	MIROC-ESM	IPSL-CM5A-LR	GFDL-CM3	HadGEM2-ES
El Morro National Monument	MIROC-ESM-CHEM	MIROC-ESM	IPSL-CM5A-LR	GFDL-CM3	HadGEM2-ES
Eleanor Roosevelt National Historic Site	MIROC-ESM-CHEM	MIROC-ESM	GFDL-CM3	MIROC5	IPSL-CM5A-LR
Everglades National Park	GFDL-CM3	MIROC-ESM-CHEM	MIROC-ESM	HadGEM2-ES	IPSL-CM5A-LR
Federal Hall National Memorial	MIROC-ESM	MIROC-ESM-CHEM	GFDL-CM3	MIROC5	HadGEM2-ES
Fire Island National Seashore	MIROC-ESM-CHEM	MIROC-ESM	GFDL-CM3	MIROC5	IPSL-CM5A-LR
Florissant Fossil Beds National Monument	MIROC-ESM-CHEM	IPSL-CM5A-LR	GFDL-CM3	HadGEM2-AO	ACCESS1-0
Fort Bowie National Historic Site	MIROC-ESM-CHEM	IPSL-CM5A-LR	MIROC-ESM	HadGEM2-ES	HadGEM2-CC
Fossil Butte National Monument	MIROC-ESM-CHEM	MIROC-ESM	HadGEM2-AO	GFDL-CM3	MIROC5
Fort Caroline National Memorial	MIROC-ESM-CHEM	MIROC-ESM	GFDL-CM3	HadGEM2-ES	IPSL-CM5A-LR
Fort Donelson National Battlefield	HadGEM2-ES	MIROC-ESM	ACCESS1-0	HadGEM2-AO	MIROC-ESM-CHEM
Fort Laramie National Historic Site	HadGEM2-AO	ACCESS1-0	MIROC-ESM-CHEM	MIROC-ESM	IPSL-CM5A-LR
Fort Larned National Historic Site	MIROC-ESM-CHEM	HadGEM2-ES	HadGEM2-AO	ACCESS1-0	MIROC5

Park	Model 1	Model 2	Model 3	Model 4	Model 5
Fort Matanzas National Monument	MIROC-ESM-CHEM	MIROC-ESM	GFDL-CM3	IPSL-CM5A-LR	HadGEM2-ES
Fort McHenry National Monument and Historic Shrine	MIROC-ESM	MIROC-ESM-CHEM	HadGEM2-ES	GFDL-CM3	ACCESS1-0
Fort Necessity National Battlefield	GFDL-CM3	MIROC-ESM	HadGEM2-ES	ACCESS1-0	MIROC-ESM-CHEM
Fort Point National Historic Site	MIROC-ESM	MIROC-ESM-CHEM	IPSL-CM5A-LR	HadGEM2-AO	HadGEM2-ES
Fort Raleigh National Historical Site	GFDL-CM3	MIROC-ESM-CHEM	MIROC-ESM	IPSL-CM5A-LR	HadGEM2-ES
Fort Scott National Historic Site	MIROC-ESM-CHEM	ACCESS1-0	MIROC-ESM	MIROC5	HadGEM2-ES
Fort Smith National Historic Site	MIROC-ESM-CHEM	ACCESS1-0	MIROC5	HadGEM2-ES	HadGEM2-AO
Fort Stanwix National Monument	MIROC-ESM	MIROC-ESM-CHEM	GFDL-CM3	MIROC5	IPSL-CM5A-LR
Fort Sumter National Monument	MIROC-ESM	GFDL-CM3	MIROC-ESM-CHEM	IPSL-CM5A-LR	HadGEM2-ES
Fort Union National Monument	MIROC-ESM-CHEM	IPSL-CM5A-LR	GFDL-CM3	MIROC-ESM	HadGEM2-ES
Fort Union Trading Post National Historic Site	MIROC-ESM	MIROC-ESM-CHEM	ACCESS1-0	HadGEM2-AO	HadGEM2-ES
Fort Vancouver National Historic Site	HadGEM2-AO	MIROC-ESM-CHEM	MIROC-ESM	IPSL-CM5A-LR	HadGEM2-ES
Fort Washington Park	MIROC-ESM	HadGEM2-ES	GFDL-CM3	MIROC-ESM-CHEM	ACCESS1-0
Friendship Hill National Historic Site	GFDL-CM3	MIROC-ESM	HadGEM2-ES	ACCESS1-0	MIROC-ESM-CHEM

Park	Model 1	Model 2	Model 3	Model 4	Model 5
Fredericksburg and Spotsylvania National Military Park	MIROC-ESM	GFDL-CM3	HadGEM2-ES	MIROC-ESM-CHEM	ACCESS1-0
Gateway National Recreation Area	MIROC-ESM-CHEM	MIROC-ESM	GFDL-CM3	MIROC5	IPSL-CM5A-LR
General Grant National Memorial	MIROC-ESM-CHEM	MIROC-ESM	GFDL-CM3	MIROC5	IPSL-CM5A-LR
Gettysburg National Military Park	MIROC-ESM	GFDL-CM3	HadGEM2-ES	MIROC5	MIROC-ESM-CHEM
George Washington Birthplace National Monument	MIROC-ESM	MIROC-ESM-CHEM	GFDL-CM3	ACCESS1-0	HadGEM2-ES
Gila Cliff Dwellings National Monument	MIROC-ESM-CHEM	MIROC-ESM	IPSL-CM5A-LR	HadGEM2-ES	HadGEM2-CC
Glacier National Park	MIROC-ESM	MIROC-ESM-CHEM	HadGEM2-AO	IPSL-CM5A-LR	HadGEM2-ES
Glacier Bay National Park & Preserve	MIROC-ESM-CHEM	IPSL-CM5A-LR	MPI-ESM-LR	MIROC-ESM	CCSM4
Glen Canyon National Recreation Area	MIROC-ESM-CHEM	MIROC-ESM	HadGEM2-AO	GFDL-CM3	IPSL-CM5A-LR
Golden Gate National Recreation Area	MIROC-ESM-CHEM	MIROC-ESM	IPSL-CM5A-LR	HadGEM2-ES	HadGEM2-AO
Governors Island National Monument	MIROC-ESM	MIROC-ESM-CHEM	GFDL-CM3	MIROC5	HadGEM2-ES
Golden Spike National Historic Site	MIROC-ESM-CHEM	MIROC-ESM	HadGEM2-AO	GFDL-CM3	ACCESS1-0
Great Basin National Park	MIROC-ESM-CHEM	MIROC-ESM	IPSL-CM5A-LR	HadGEM2-AO	HadGEM2-ES
Grand Canyon National Park	MIROC-ESM-CHEM	MIROC-ESM	HadGEM2-AO	IPSL-CM5A-LR	HadGEM2-ES

Park	Model 1	Model 2	Model 3	Model 4	Model 5
Greenbelt Park	MIROC-ESM	MIROC-ESM-CHEM	HadGEM2-ES	GFDL-CM3	ACCESS1-0
Grant-Kohrs Ranch National Historic Site	MIROC-ESM-CHEM	HadGEM2-AO	MIROC-ESM	IPSL-CM5A-LR	HadGEM2-ES
Grand Portage National Monument	MIROC-ESM	MIROC-ESM-CHEM	GFDL-CM3	HadGEM2-ES	ACCESS1-0
Great Sand Dunes National Park	GFDL-CM3	MIROC-ESM-CHEM	IPSL-CM5A-LR	ACCESS1-0	HadGEM2-ES
Great Smoky Mountains National Park	HadGEM2-ES	GFDL-CM3	MIROC-ESM	HadGEM2-CC	ACCESS1-0
Grand Teton National Park	MIROC-ESM-CHEM	MIROC-ESM	IPSL-CM5A-LR	GFDL-CM3	HadGEM2-AO
Guilford Courthouse National Military Park	HadGEM2-ES	HadGEM2-CC	GFDL-CM3	MIROC-ESM	ACCESS1-0
Gulf Islands National Seashore	MIROC-ESM-CHEM	MIROC-ESM	HadGEM2-ES	GFDL-CM3	IPSL-CM5A-LR
Guadalupe Mountains National Park	IPSL-CM5A-LR	MIROC-ESM-CHEM	HadGEM2-ES	HadGEM2-CC	GFDL-CM3
George Washington Carver National Monument	MIROC-ESM-CHEM	ACCESS1-0	MIROC-ESM	HadGEM2-AO	MIROC5
George Washington Memorial Parkway	MIROC-ESM	HadGEM2-ES	GFDL-CM3	MIROC-ESM-CHEM	ACCESS1-0
Harpers Ferry National Historical Park	MIROC-ESM	GFDL-CM3	HadGEM2-ES	MIROC-ESM-CHEM	ACCESS1-0
Hagerman Fossil Beds National Monument	MIROC-ESM-CHEM	MIROC-ESM	HadGEM2-AO	ACCESS1-0	HadGEM2-ES
Hamilton Grange National Memorial	MIROC-ESM-CHEM	MIROC-ESM	GFDL-CM3	MIROC5	IPSL-CM5A-LR
Haleakala National Park	IPSL-CM5A-LR	HadGEM2-ES	GFDL-CM3	HadGEM2-CC	HadGEM2-AO

Park	Model 1	Model 2	Model 3	Model 4	Model 5
Hawai'i Volcanoes National Park	IPSL-CM5A-LR	HadGEM2-ES	GFDL-CM3	HadGEM2-AO	HadGEM2-CC
Herbert Hoover National Historic Site	MIROC-ESM	ACCESS1-0	MIROC-ESM-CHEM	HadGEM2-ES	HadGEM2-AO
Horseshoe Bend National Military Park	MIROC-ESM-CHEM	HadGEM2-ES	HadGEM2-CC	ACCESS1-0	IPSL-CM5A-LR
Hopewell Culture National Historical Park	HadGEM2-ES	ACCESS1-0	GFDL-CM3	MIROC-ESM	MIROC-ESM-CHEM
Home of Franklin D. Roosevelt National Historic Site	MIROC-ESM-CHEM	MIROC-ESM	GFDL-CM3	MIROC5	IPSL-CM5A-LR
Hopewell Furnace National Historic Site	MIROC-ESM	MIROC-ESM-CHEM	HadGEM2-ES	GFDL-CM3	MIROC5
Homestead National Monument	ACCESS1-0	MIROC-ESM-CHEM	HadGEM2-AO	MIROC-ESM	MIROC5
Hot Springs National Park	MIROC-ESM-CHEM	HadGEM2-ES	ACCESS1-0	MIROC5	HadGEM2-AO
Hovenweep National Monument	MIROC-ESM-CHEM	MIROC-ESM	GFDL-CM3	HadGEM2-AO	IPSL-CM5A-LR
Harry S. Truman National Historic Site	ACCESS1-0	MIROC-ESM-CHEM	MIROC-ESM	HadGEM2-AO	HadGEM2-ES
Hubbell Trading Post National Historic Site	MIROC-ESM-CHEM	MIROC-ESM	IPSL-CM5A-LR	GFDL-CM3	HadGEM2-ES
Independence National Historical Park	MIROC-ESM	MIROC-ESM-CHEM	GFDL-CM3	MIROC5	IPSL-CM5A-LR
Indiana Dunes National Lakeshore	MIROC-ESM	MIROC-ESM-CHEM	ACCESS1-0	HadGEM2-ES	HadGEM2-AO
Isle Royale National Park	MIROC-ESM	MIROC-ESM-CHEM	GFDL-CM3	HadGEM2-ES	ACCESS1-0

Park	Model 1	Model 2	Model 3	Model 4	Model 5
James A Garfield National Historic Site	MIROC-ESM	MIROC-ESM-CHEM	HadGEM2-ES	ACCESS1-0	HadGEM2-CC
Jewel Cave National Monument	HadGEM2-AO	ACCESS1-0	MIROC-ESM-CHEM	MIROC-ESM	HadGEM2-ES
Jefferson National Expansion Memorial National Memorial	MIROC-ESM-CHEM	MIROC-ESM	ACCESS1-0	HadGEM2-AO	HadGEM2-ES
Jimmy Carter National Historic Site	MIROC-ESM-CHEM	HadGEM2-ES	HadGEM2-CC	ACCESS1-0	GFDL-CM3
John Day Fossil Beds National Monument	HadGEM2-AO	MIROC-ESM	MIROC-ESM-CHEM	HadGEM2-ES	IPSL-CM5A-LR
John D. Rockefeller, Jr. Memorial Parkway	MIROC-ESM-CHEM	MIROC-ESM	IPSL-CM5A-LR	HadGEM2-AO	GFDL-CM3
John Fitzgerald Kennedy National Historic Site	MIROC-ESM-CHEM	MIROC-ESM	GFDL-CM3	MIROC5	HadGEM2-ES
Johnstown Flood National Memorial	GFDL-CM3	MIROC-ESM	MIROC-ESM-CHEM	HadGEM2-ES	ACCESS1-0
Joshua Tree National Park	GFDL-CM3	HadGEM2-AO	IPSL-CM5A-LR	HadGEM2-ES	HadGEM2-CC
Katmai National Park & Preserve	HadGEM2-ES	HadGEM2-AO	ACCESS1-0	GFDL-CM3	HadGEM2-CC
Kenai Fjords National Park	HadGEM2-AO	GFDL-CM3	HadGEM2-ES	BCC-CSM1-1	IPSL-CM5A-LR
Kennesaw Mountain National Battlefield Park	HadGEM2-ES	HadGEM2-CC	HadGEM2-AO	MIROC-ESM	MIROC-ESM-CHEM
Kings Canyon National Park	MIROC-ESM	MIROC-ESM-CHEM	IPSL-CM5A-LR	GFDL-CM3	HadGEM2-AO
Kings Mountain National Military Park	HadGEM2-ES	HadGEM2-CC	GFDL-CM3	ACCESS1-0	MIROC-ESM
Klondike Gold Rush AK National Historical Park	MIROC-ESM-CHEM	MIROC-ESM	GFDL-CM3	IPSL-CM5A-LR	HadGEM2-ES

Park	Model 1	Model 2	Model 3	Model 4	Model 5
Klondike Gold Rush WA National Historical Park	HadGEM2-AO	HadGEM2-ES	HadGEM2-CC	IPSL-CM5A-LR	MIROC-ESM
Knife River Indian Villages National Historic Site	MIROC-ESM	MIROC-ESM-CHEM	ACCESS1-0	HadGEM2-ES	HadGEM2-AO
Korean War Veterans Memorial National Memorial	MIROC-ESM	HadGEM2-ES	GFDL-CM3	MIROC-ESM-CHEM	ACCESS1-0
Lava Beds National Monument	HadGEM2-AO	HadGEM2-ES	IPSL-CM5A-LR	GFDL-CM3	HadGEM2-CC
Lake Chelan National Recreation Area	HadGEM2-AO	HadGEM2-ES	IPSL-CM5A-LR	HadGEM2-CC	MIROC-ESM-CHEM
Lake Clark National Park & Preserve	GFDL-CM3	HadGEM2-ES	HadGEM2-AO	ACCESS1-0	MPI-ESM-LR
Lake Mead National Recreation Area	MIROC-ESM	MIROC-ESM-CHEM	HadGEM2-AO	IPSL-CM5A-LR	HadGEM2-ES
Lake Meredith National Recreation Area	MIROC-ESM-CHEM	IPSL-CM5A-LR	HadGEM2-ES	GFDL-CM3	ACCESS1-0
Lake Roosevelt National Recreation Area	HadGEM2-AO	MIROC-ESM-CHEM	MIROC-ESM	HadGEM2-ES	IPSL-CM5A-LR
Lassen Volcanic National Park	HadGEM2-AO	HadGEM2-ES	IPSL-CM5A-LR	HadGEM2-CC	GFDL-CM3
Lewis and Clark National Historical Trail	IPSL-CM5A-LR	HadGEM2-AO	MIROC-ESM-CHEM	MIROC-ESM	HadGEM2-ES
Little Bighorn Battlefield National Monument	HadGEM2-AO	MIROC-ESM	MIROC-ESM-CHEM	ACCESS1-0	HadGEM2-ES
Lincoln Boyhood National Memorial	MIROC-ESM	HadGEM2-ES	ACCESS1-0	MIROC-ESM-CHEM	HadGEM2-AO
Lincoln Home National Historic Site	MIROC-ESM	MIROC-ESM-CHEM	ACCESS1-0	HadGEM2-AO	HadGEM2-ES
Lincoln Memorial National Memorial	MIROC-ESM	HadGEM2-ES	GFDL-CM3	MIROC-ESM-CHEM	ACCESS1-0

Park	Model 1	Model 2	Model 3	Model 4	Model 5
Little River Canyon National Preserve	HadGEM2-ES	HadGEM2-CC	ACCESS1-0	MIROC-ESM	GFDL-CM3
Longfellow National Historic Site	MIROC-ESM-CHEM	MIROC-ESM	GFDL-CM3	MIROC5	HadGEM2-ES
Lowell National Historical Park	MIROC-ESM-CHEM	MIROC-ESM	GFDL-CM3	MIROC5	HadGEM2-ES
Lyndon Baines Johnson Memorial Grove on the Potomac National Memorial	MIROC-ESM	HadGEM2-ES	GFDL-CM3	MIROC-ESM-CHEM	ACCESS1-0
Mammoth Cave National Park	HadGEM2-ES	ACCESS1-0	MIROC-ESM	HadGEM2-AO	HadGEM2-CC
Martin Luther King, Jr. National Historic Site	HadGEM2-ES	HadGEM2-CC	HadGEM2-AO	MIROC-ESM	MIROC-ESM-CHEM
Manassas National Battlefield Park	MIROC-ESM	GFDL-CM3	HadGEM2-ES	MIROC-ESM-CHEM	ACCESS1-0
Manzanar National Historic Site	MIROC-ESM	MIROC-ESM-CHEM	IPSL-CM5A-LR	HadGEM2-ES	HadGEM2-AO
Martin Van Buren National Historic Site	MIROC-ESM-CHEM	GFDL-CM3	MIROC-ESM	MIROC5	IPSL-CM5A-LR
Mesa Verde National Park	MIROC-ESM-CHEM	GFDL-CM3	MIROC-ESM	HadGEM2-AO	IPSL-CM5A-LR
Minute Man National Historical Park	MIROC-ESM-CHEM	MIROC-ESM	GFDL-CM3	MIROC5	HadGEM2-ES
Minuteman Missile National Historic Site	HadGEM2-AO	ACCESS1-0	MIROC-ESM	MIROC-ESM-CHEM	HadGEM2-ES
Missouri National Recreation River	MIROC-ESM	ACCESS1-0	HadGEM2-AO	MIROC-ESM-CHEM	HadGEM2-ES
Montezuma Castle National Monument	MIROC-ESM-CHEM	MIROC-ESM	IPSL-CM5A-LR	HadGEM2-ES	HadGEM2-AO

Park	Model 1	Model 2	Model 3	Model 4	Model 5
Monocacy National Battlefield	MIROC-ESM	HadGEM2-ES	GFDL-CM3	MIROC-ESM-CHEM	ACCESS1-0
Mount Rainier National Park	HadGEM2-AO	HadGEM2-ES	HadGEM2-CC	MIROC-ESM	MIROC-ESM-CHEM
Morristown National Historical Park	MIROC-ESM	MIROC-ESM-CHEM	GFDL-CM3	HadGEM2-ES	MIROC5
Mount Rushmore National Memorial	HadGEM2-AO	ACCESS1-0	MIROC-ESM	MIROC-ESM-CHEM	HadGEM2-ES
Muir Woods National Monument	MIROC-ESM-CHEM	MIROC-ESM	IPSL-CM5A-LR	HadGEM2-ES	HadGEM2-AO
Natural Bridges National Monument	MIROC-ESM-CHEM	MIROC-ESM	GFDL-CM3	HadGEM2-AO	IPSL-CM5A-LR
National Capital combined Park	MIROC-ESM	HadGEM2-ES	GFDL-CM3	MIROC-ESM-CHEM	ACCESS1-0
Natchez National Historical Park	MIROC-ESM-CHEM	MIROC5	HadGEM2-ES	HadGEM2-CC	HadGEM2-AO
Natchez Trace Parkway and National Scenic Trail	HadGEM2-ES	MIROC-ESM-CHEM	ACCESS1-0	HadGEM2-CC	HadGEM2-AO
Navajo National Monument	MIROC-ESM-CHEM	MIROC-ESM	HadGEM2-AO	GFDL-CM3	IPSL-CM5A-LR
Nez Perce National Historical Park	HadGEM2-AO	MIROC-ESM-CHEM	MIROC-ESM	HadGEM2-ES	IPSL-CM5A-LR
New River Gorge National River	GFDL-CM3	HadGEM2-ES	ACCESS1-0	HadGEM2-CC	MIROC-ESM
Nicodemus National Historic Site	MIROC-ESM-CHEM	HadGEM2-ES	HadGEM2-AO	ACCESS1-0	MIROC5
Niobrara National Scenic River	ACCESS1-0	MIROC-ESM	HadGEM2-AO	HadGEM2-ES	MIROC-ESM-CHEM

Park	Model 1	Model 2	Model 3	Model 4	Model 5
Ninety Six National Historic Site	HadGEM2-ES	HadGEM2-CC	MIROC-ESM	GFDL-CM3	ACCESS1-0
North Cascades National Park	HadGEM2-AO	HadGEM2-ES	IPSL-CM5A-LR	HadGEM2-CC	MIROC-ESM-CHEM
Obed Wild and Scenic River	HadGEM2-ES	GFDL-CM3	HadGEM2-CC	ACCESS1-0	MIROC-ESM
Olympic National Park	HadGEM2-AO	IPSL-CM5A-LR	HadGEM2-ES	MIROC-ESM	HadGEM2-CC
Oregon Caves National Monument	IPSL-CM5A-LR	HadGEM2-ES	HadGEM2-AO	MIROC-ESM-CHEM	HadGEM2-CC
Ozark National Scenic Riverway	MIROC-ESM-CHEM	MIROC-ESM	ACCESS1-0	HadGEM2-ES	HadGEM2-AO
Palo Alto Battlefield National Historic Site	IPSL-CM5A-LR	MIROC-ESM-CHEM	GFDL-CM3	HadGEM2-ES	HadGEM2-CC
Pennsylvania Avenue National Historic Site National Historic Site	MIROC-ESM	HadGEM2-ES	GFDL-CM3	MIROC-ESM-CHEM	ACCESS1-0
Padre Island National Seashore	IPSL-CM5A-LR	MIROC-ESM-CHEM	GFDL-CM3	MIROC-ESM	HadGEM2-ES
Pecos National Historic Park	MIROC-ESM-CHEM	IPSL-CM5A-LR	MIROC-ESM	GFDL-CM3	HadGEM2-ES
Petrified Forest National Park	MIROC-ESM-CHEM	MIROC-ESM	IPSL-CM5A-LR	GFDL-CM3	HadGEM2-ES
Pea Ridge National Military Park	MIROC-ESM-CHEM	ACCESS1-0	MIROC-ESM	HadGEM2-ES	HadGEM2-AO
Petersburg National Battlefield	MIROC-ESM	GFDL-CM3	ACCESS1-0	MIROC-ESM-CHEM	HadGEM2-ES
Petroglyph National Monument	MIROC-ESM-CHEM	MIROC-ESM	IPSL-CM5A-LR	GFDL-CM3	HadGEM2-ES
Perry's Victory and International Peace Memorial	MIROC-ESM	MIROC-ESM-CHEM	HadGEM2-ES	ACCESS1-0	MIROC5

Park	Model 1	Model 2	Model 3	Model 4	Model 5
Pipestone National Monument	MIROC-ESM	ACCESS1-0	MIROC-ESM-CHEM	HadGEM2-AO	HadGEM2-ES
Pictured Rocks National Lakeshore	MIROC-ESM	MIROC-ESM-CHEM	GFDL-CM3	HadGEM2-ES	ACCESS1-0
Piscataway Park	MIROC-ESM	HadGEM2-ES	GFDL-CM3	MIROC-ESM-CHEM	ACCESS1-0
Pipe Spring National Monument	MIROC-ESM-CHEM	MIROC-ESM	HadGEM2-AO	HadGEM2-ES	IPSL-CM5A-LR
Point Reyes National Seashore	MIROC-ESM-CHEM	MIROC-ESM	IPSL-CM5A-LR	HadGEM2-ES	HadGEM2-AO
Prince William Forest Park	MIROC-ESM	HadGEM2-ES	GFDL-CM3	MIROC-ESM-CHEM	ACCESS1-0
Rainbow Bridge National Monument	MIROC-ESM-CHEM	MIROC-ESM	HadGEM2-AO	GFDL-CM3	IPSL-CM5A-LR
Redwood National Park	HadGEM2-AO	IPSL-CM5A-LR	HadGEM2-ES	MIROC-ESM-CHEM	HadGEM2-CC
Richmond National Battlefield Park	MIROC-ESM	GFDL-CM3	MIROC-ESM-CHEM	ACCESS1-0	HadGEM2-ES
Rock Creek Park Park	MIROC-ESM	HadGEM2-ES	GFDL-CM3	MIROC-ESM-CHEM	ACCESS1-0
Ross Lake National Recreation Area	HadGEM2-AO	IPSL-CM5A-LR	HadGEM2-ES	HadGEM2-CC	MIROC-ESM-CHEM
Rocky Mountain National Park	MIROC-ESM-CHEM	HadGEM2-AO	ACCESS1-0	IPSL-CM5A-LR	GFDL-CM3
Roger Williams National Memorial	MIROC-ESM-CHEM	MIROC-ESM	GFDL-CM3	MIROC5	HadGEM2-ES
Russell Cave National Monument	HadGEM2-ES	HadGEM2-CC	MIROC-ESM	GFDL-CM3	ACCESS1-0

Park	Model 1	Model 2	Model 3	Model 4	Model 5
San Antonio Missions National Historic Park	IPSL-CM5A-LR	MIROC-ESM-CHEM	GFDL-CM3	HadGEM2-ES	HadGEM2-AO
Saint Croix National Scenic River	MIROC-ESM	MIROC-ESM-CHEM	ACCESS1-0	HadGEM2-ES	HadGEM2-AO
San Francisco Maritime National Historical Park	MIROC-ESM	MIROC-ESM-CHEM	IPSL-CM5A-LR	HadGEM2-AO	HadGEM2-ES
Saint-Gaudens National Historic Site	MIROC-ESM-CHEM	GFDL-CM3	MIROC-ESM	MIROC5	ACCESS1-0
Sagamore Hill National Historic Site	MIROC-ESM-CHEM	MIROC-ESM	GFDL-CM3	MIROC5	IPSL-CM5A-LR
Saugus Iron Works National Historic Site	MIROC-ESM-CHEM	MIROC-ESM	GFDL-CM3	MIROC5	HadGEM2-ES
San Juan National Historical Site	IPSL-CM5A-LR	GFDL-CM3	MIROC-ESM-CHEM	HadGEM2-ES	MIROC-ESM
Salem Maritime National Historic Site	MIROC-ESM-CHEM	MIROC-ESM	GFDL-CM3	MIROC5	HadGEM2-ES
Salinas Pueblo Missions National Monument	MIROC-ESM-CHEM	MIROC-ESM	IPSL-CM5A-LR	HadGEM2-ES	GFDL-CM3
Saratoga National Historical Park	MIROC-ESM-CHEM	GFDL-CM3	MIROC-ESM	MIROC5	ACCESS1-0
Scotts Bluff National Monument	HadGEM2-AO	ACCESS1-0	MIROC-ESM-CHEM	MIROC-ESM	IPSL-CM5A-LR
Sequoia National Park	MIROC-ESM	MIROC-ESM-CHEM	IPSL-CM5A-LR	HadGEM2-AO	HadGEM2-ES
Shenandoah National Park	MIROC-ESM	GFDL-CM3	HadGEM2-ES	ACCESS1-0	MIROC-ESM-CHEM
Shiloh National Battlefield	HadGEM2-ES	HadGEM2-AO	ACCESS1-0	MIROC-ESM	MIROC-ESM-CHEM

Park	Model 1	Model 2	Model 3	Model 4	Model 5
Sitka National Historical Park	MIROC-ESM-CHEM	IPSL-CM5A-LR	MPI-ESM-LR	MIROC-ESM	HadGEM2-ES
Sleeping Bear Dunes National Lakeshore	MIROC-ESM	MIROC-ESM-CHEM	HadGEM2-ES	ACCESS1-0	GFDL-CM3
Springfield Armory National Historic Site	MIROC-ESM-CHEM	MIROC-ESM	GFDL-CM3	MIROC5	HadGEM2-ES
Steamtown National Historic Site	MIROC-ESM	MIROC-ESM-CHEM	GFDL-CM3	HadGEM2-ES	ACCESS1-0
Statue Of Liberty National Monument	MIROC-ESM	MIROC-ESM-CHEM	GFDL-CM3	MIROC5	HadGEM2-ES
Stones River National Battlefield	HadGEM2-ES	ACCESS1-0	MIROC-ESM	HadGEM2-CC	HadGEM2-AO
Sunset Crater Volcano National Monument	MIROC-ESM-CHEM	MIROC-ESM	IPSL-CM5A-LR	HadGEM2-ES	GFDL-CM3
Tallgrass Prairie National Preserve	MIROC-ESM-CHEM	ACCESS1-0	MIROC-ESM	HadGEM2-AO	MIROC5
Theodore Roosevelt Island National Memorial	MIROC-ESM	HadGEM2-ES	GFDL-CM3	MIROC-ESM-CHEM	ACCESS1-0
Theodore Roosevelt National Park	MIROC-ESM	ACCESS1-0	MIROC-ESM-CHEM	HadGEM2-AO	HadGEM2-ES
Timpanogos Cave National Monument	MIROC-ESM-CHEM	MIROC-ESM	HadGEM2-AO	GFDL-CM3	ACCESS1-0
Tumacacori National Historical Park	IPSL-CM5A-LR	MIROC-ESM-CHEM	HadGEM2-ES	HadGEM2-CC	GFDL-CM3
Tuzigoot National Monument	MIROC-ESM-CHEM	MIROC-ESM	IPSL-CM5A-LR	GFDL-CM3	HadGEM2-ES
Ulysses S. Grant National Historic Site	MIROC-ESM-CHEM	MIROC-ESM	ACCESS1-0	HadGEM2-AO	HadGEM2-ES

Park	Model 1	Model 2	Model 3	Model 4	Model 5
Upper Delaware Scenic and Recreational River	MIROC-ESM	MIROC-ESM-CHEM	GFDL-CM3	HadGEM2-ES	ACCESS1-0
Valley Forge National Historical Park	MIROC-ESM	MIROC-ESM-CHEM	GFDL-CM3	HadGEM2-ES	MIROC5
World War II Valor in the Pacific National Monument National Monument	IPSL-CM5A-LR	GFDL-CM3	HadGEM2-ES	HadGEM2-AO	HadGEM2-CC
Vanderbilt Mansion National Historic Site	MIROC-ESM-CHEM	MIROC-ESM	GFDL-CM3	MIROC5	IPSL-CM5A-LR
Vicksburg National Military Park	MIROC-ESM-CHEM	HadGEM2-ES	MIROC5	HadGEM2-CC	HadGEM2-AO
Virgin Islands National Park	IPSL-CM5A-LR	GFDL-CM3	MIROC-ESM	MIROC-ESM-CHEM	HadGEM2-ES
Vietnam Veterans Memorial National Memorial	MIROC-ESM	HadGEM2-ES	GFDL-CM3	MIROC-ESM-CHEM	ACCESS1-0
Voyageurs National Park	MIROC-ESM	MIROC-ESM-CHEM	GFDL-CM3	ACCESS1-0	HadGEM2-ES
Washita Battlefield National Historic Site	MIROC-ESM-CHEM	IPSL-CM5A-LR	HadGEM2-ES	ACCESS1-0	MIROC-ESM
Walnut Canyon National Monument	MIROC-ESM-CHEM	MIROC-ESM	IPSL-CM5A-LR	HadGEM2-ES	GFDL-CM3
Weir Farm National Historic Site	MIROC-ESM-CHEM	MIROC-ESM	GFDL-CM3	MIROC5	IPSL-CM5A-LR
Whiskeytown National Recreation Area	HadGEM2-ES	HadGEM2-AO	IPSL-CM5A-LR	HadGEM2-CC	MIROC-ESM-CHEM
Whitman Mission National Historic Site	HadGEM2-AO	MIROC-ESM-CHEM	MIROC-ESM	IPSL-CM5A-LR	HadGEM2-ES
White Sands National Monument	MIROC-ESM-CHEM	IPSL-CM5A-LR	MIROC-ESM	HadGEM2-ES	HadGEM2-CC

Park	Model 1	Model 2	Model 3	Model 4	Model 5
Wind Cave National Park	HadGEM2-AO	ACCESS1-0	MIROC-ESM-CHEM	MIROC-ESM	HadGEM2-ES
Wilson's Creek National Battlefield	MIROC-ESM-CHEM	ACCESS1-0	MIROC-ESM	HadGEM2-ES	HadGEM2-AO
William Howard Taft National Historic Site	HadGEM2-ES	MIROC-ESM	MIROC-ESM-CHEM	ACCESS1-0	HadGEM2-AO
Women's Rights National Historical Park	MIROC-ESM	MIROC-ESM-CHEM	GFDL-CM3	HadGEM2-ES	ACCESS1-0
Wolf Trap National Park for the Performing Arts	MIROC-ESM	HadGEM2-ES	GFDL-CM3	MIROC-ESM-CHEM	ACCESS1-0
Wright Brothers National Memorial	MIROC-ESM-CHEM	GFDL-CM3	MIROC-ESM	IPSL-CM5A-LR	HadGEM2-ES
Wrangell-St. Elias National Park & Preserve	GFDL-CM3	MIROC-ESM-CHEM	HadGEM2-ES	IPSL-CM5A-LR	BCC-CSM1-1
Wupatki National Monument	MIROC-ESM-CHEM	MIROC-ESM	IPSL-CM5A-LR	HadGEM2-ES	HadGEM2-AO
World War II Memorial National Memorial	MIROC-ESM	HadGEM2-ES	GFDL-CM3	MIROC-ESM-CHEM	ACCESS1-0
Yellowstone National Park	MIROC-ESM-CHEM	MIROC-ESM	IPSL-CM5A-LR	HadGEM2-AO	GFDL-CM3
Yosemite National Park	MIROC-ESM	MIROC-ESM-CHEM	IPSL-CM5A-LR	GFDL-CM3	HadGEM2-AO
Zion National Park	MIROC-ESM-CHEM	MIROC-ESM	HadGEM2-AO	IPSL-CM5A-LR	HadGEM2-ES
