# Supplementary material for: Protected Area Tourism in a Changing Climate: Will Visitation at US National Parks Warm Up or Overheat?
Source: PLoS One. 2015 Jun 17;10(6):e0128226. doi: 10.1371/journal.pone.0128226 (PMC4470629; doi:10.1371/journal.pone.0128226)
Supplement: S3 Table — The third-order polynomial model was the best-fit model (lowest BIC). Results shown are based on a model that includes park name as a categorical explanatory variable and a quasibinomial error distribution to account for overdispersion, and has 3737 degrees of freedom. (PDF) [file pone.0128226.s008.pdf]

S3 Table. **Generalized linear model output for historical (1979-2013) U.S. National Park System monthly visitation relationship to monthly mean temperature.** The third-order polynomial model was the best-fit model (lowest BIC). Results shown are based on a model that includes park name as a categorical explanatory variable and a quasibinomial error distribution to account for overdispersion, and has 3737 degrees of freedom.

Parameter	Estimate	Std. Error	z value	p value
Intercept	-3.90E+00	1.27E-01	-30.598	< 0.0001
Temperature	1.55E-01	4.39E-03	35.189	< 0.0001
Temperature^2	-2.29E-03	3.78E-04	-6.06	< 0.0001
Temperature^3	-3.18E-05	9.67E-06	-3.283	0.001
				R^2 = 0.69

Model	ΔBIC
3rd-order polynomial	0
2nd-order polynomial	118
Temperature	14308
Null model	86919
