# Supplementary material for: Protected Area Tourism in a Changing Climate: Will Visitation at US National Parks Warm Up or Overheat?
Source: PLoS One. 2015 Jun 17;10(6):e0128226. doi: 10.1371/journal.pone.0128226 (PMC4470629; doi:10.1371/journal.pone.0128226)
Supplement: S4 Table — Output shown from the best-fit (lowest-BIC) model among four models of increasing complexity (a null model [‘Null’], a model including monthly temperature as the single explanatory variable [‘1st-order polynomial’], the second-order polynomial equation of temperature [‘2nd-order polynomial’], and the third-order polynomial equation of temperature [‘3rd-order polynomial’]). (PDF) [file pone.0128226.s009.pdf]

S4. Table. **Linear model relationship between historical monthly visitation and temperature at individual U.S. national parks.** Output shown from the best-fit (lowest-BIC) model among four models of increasing complexity (a null model ['Null'], a model including monthly temperature as the single explanatory variable ['1st-order polynomial'], the second-order polynomial equation of temperature ['2nd-order polynomial'], and the third-order polynomial equation of temperature ['3rd-order polynomial'])

Park	Best-fit model	F-value	P-value	adj. R^2	Intercept	Temperature	$Temperature^2$	$Temperature^3$
Abraham Lincoln Birthplace National Historic Site	2nd-order polynomial	141.8	<0.0001	0.96	3832.94	515.71	36.97	
Acadia National Park	2nd-order polynomial	77.2	<0.0001	0.93	39221.16	9351.89	1109.86	
Adams National Historical Park	1st-order polynomial	132.1	<0.0001	0.92	1678.92	701.45		
Agate Fossil Beds National Monument	2nd-order polynomial	255.7	<0.0001	0.98	160.96	31.78	4.67	
Allegheny Portage Railroad National Historic Site	2nd-order polynomial	157.9	<0.0001	0.97	5112.65	348.26	6.30	
Amistad National Recreation Area	2nd-order polynomial	4.6	0.0429	0.39	-54498.30	14716.77	-292.94	
Andersonville National Historical Site	2nd-order polynomial	8.1	0.0099	0.56	-8370.91	2253.57	-54.76	
Andrew Johnson National Historic Site	1st-order polynomial	10.7	0.0085	0.47	3141.72	158.17		
Antietam National Battlefield	1st-order polynomial	50.3	<0.0001	0.82	8778.35	1529.26		
Apostle Islands National Lakeshore	3rd-order polynomial	62.8	<0.0001	0.94	1739.52	-225.66	21.66	5.34
Appomattox Court House National Historical Park	1st-order polynomial	100.6	<0.0001	0.9	1095.94	1320.90		
Arches National Park	3rd-order polynomial	110.2	<0.0001	0.97	13486.65	2744.15	292.19	-10.74

Park	Best-fit model	F-value	P-value	adj. R^2	Intercept	Temperature	Temperature^2	Temperature^3
Arkansas Post National Memorial	1st-order polynomial	10.6	0.0086	0.47	2515.57	97.28		
Arlington House / The Robert E. Lee National Memorial	1st-order polynomial	22	0.0009	0.66	13659.77	1916.93		
Assateague Island National Seashore	3rd-order polynomial	126.9	<0.0001	0.97	-17847.60	21123.31	-1690.14	60.78
Aztec Ruins National Monument	2nd-order polynomial	140.5	<0.0001	0.96	1375.51	182.61	6.17	
Badlands National Park	3rd-order polynomial	260.2	<0.0001	0.99	10892.75	434.65	227.00	9.19
Bandelier National Monument	2nd-order polynomial	80.4	<0.0001	0.94	8513.07	2068.42	-38.09	
Bent's Old Fort National Historic Site	2nd-order polynomial	46.6	<0.0001	0.89	987.51	89.44	5.27	
Big Bend National Park	2nd-order polynomial	5.3	0.0303	0.44	-19170.24	5420.51	-142.84	
Big Cypress National Preserve	1st-order polynomial	86.9	<0.0001	0.89	162496.70	-4810.77		
Big Hole National Battlefield	2nd-order polynomial	398	<0.0001	0.99	549.40	193.21	29.62	
Big South Fork National River and Recreation Area	2nd-order polynomial	35.9	<0.0001	0.86	18516.84	5767.89	-138.57	
Big Thicket National Preserve	1st-order polynomial	10.6	0.0086	0.47	2878.59	219.66		
Bighorn Canyon National Recreation Area	3rd-order polynomial	165.6	<0.0001	0.98	14451.39	950.03	80.40	-2.17
Biscayne National Park	1st-order polynomial	7.4	0.0218	0.37	-5538.97	1677.77		

Park	Best-fit model	F-value	P-value	adj. R^2	Intercept	Temperature	$Temperature^2$	$Temperature^3$
Black Canyon of the Gunnison National Park	2nd-order polynomial	646.5	<0.0001	0.99	3345.20	413.29	91.09	
Blue Ridge Parkway	1st-order polynomial	42.5	<0.0001	0.79	411719.00	80432.46		
Bluestone National Scenic River	2nd-order polynomial	46.8	<0.0001	0.89	25.52	-70.92	25.01	
Booker T. Washington National Monument	1st-order polynomial	70.5	<0.0001	0.86	207.51	113.26		
Boston African American National Historic Site	3rd-order polynomial	72.3	<0.0001	0.95	6229.85	1995.17	-553.84	29.15
Boston National Historical Park	1st-order polynomial	155	<0.0001	0.93	60348.27	11025.08		
Brown v. Board of Education National Historic Site	1st-order polynomial	6.7	0.027	0.34	1195.47	33.13		
Bryce Canyon National Park	3rd-order polynomial	1176.4	<0.0001	0.99	16942.58	3771.64	592.48	-20.37
Buck Island Reef National Monument	1st-order polynomial	9	0.0132	0.42	17705.03	-511.00		
Buffalo National River	1st-order polynomial	33	0.0002	0.74	-11740.30	5797.65		
Cabrillo National Monument	Null				95850.15			
Canaveral National Seashore	1st-order polynomial	2.6	0.1379	0.13	45590.60	1943.18		
Cane River Creole National Historical Park	2nd-order polynomial	3	0.1026	0.26	-1006.05	318.71	-7.57	
Canyon de Chelly National Monument	1st-order polynomial	89.4	<0.0001	0.89	37245.34	2378.13		
Canyonlands National Park	3rd-order polynomial	60.2	<0.0001	0.94	5565.61	1856.55	170.83	-8.15

Park	Best-fit model	F-value	P-value	adj. R^2	Intercept	Temperature	$Temperature^2$	$Temperature^3$
Cape Cod National Seashore	2nd-order polynomial	86.1	<0.0001	0.94	127405.00	-2673.46	1539.34	
Cape Hatteras National Seashore	3rd-order polynomial	199.2	<0.0001	0.98	-132858.20	36694.21	-2054.61	52.02
Cape Lookout National Seashore	1st-order polynomial	97.9	<0.0001	0.9	-4728.08	2073.64		
Capitol Reef National Park	3rd-order polynomial	136	<0.0001	0.97	12873.04	3471.34	314.85	-17.08
Capulin Volcano National Monument	3rd-order polynomial	57.7	<0.0001	0.94	816.69	550.19	-76.87	3.57
Carl Sandburg Home National Historic Site	2nd-order polynomial	21.3	0.0004	0.79	-1094.73	564.38	-11.15	
Carlsbad Caverns National Park	3rd-order polynomial	26.4	0.0002	0.87	-72542.02	24773.43	-1797.12	42.32
Casa Grande Ruins National Monument	1st-order polynomial	12	0.0061	0.5	22010.09	-522.53		
Castillo de San Marcos National Monument	Null				56967.07			
Castle Clinton National Monument	3rd-order polynomial	83.3	<0.0001	0.96	100818.50	24295.40	-1902.65	58.59
Catoctin Mountain Park	2nd-order polynomial	41.4	<0.0001	0.88	19918.81	2744.33	-57.28	
Cedar Breaks National Monument	2nd-order polynomial	34.1	<0.0001	0.86	7608.60	1119.84	191.40	
Chaco Culture National Historical Park	2nd-order polynomial	39.4	<0.0001	0.87	1393.81	553.15	-12.27	
Chamizal National Memorial	3rd-order polynomial	22.8	0.0003	0.86	-46608.17	11595.75	-743.75	15.31

Park	Best-fit model	F-value	P-value	adj. R^2	Intercept	Temperature	Temperature^2	Temperature^3
Channel Islands National Park	1st-order polynomial	22	0.0009	0.66	-15223.50	2758.12		
Charles Pinckney National Historic Site	2nd-order polynomial	8.7	0.0079	0.58	-1220.55	404.17	-8.86	
Chattahoochee River National Recreation Area	1st-order polynomial	27.2	0.0004	0.7	109650.00	5101.92		
Chesapeake and Ohio Canal National Historic Park	2nd-order polynomial	254.3	<0.0001	0.98	104387.70	21534.55	-339.05	
Chickamauga and Chattanooga National Military Park	1st-order polynomial	113.7	<0.0001	0.91	45792.36	2279.52		
Chickasaw National Recreation Area	1st-order polynomial	51.7	<0.0001	0.82	-6774.17	7948.53		
Chiricahua National Monument	2nd-order polynomial	3.3	0.0863	0.29	-809.44	1035.17	-33.00	
Christiansted National Historic Site	1st-order polynomial	32.9	0.0002	0.74	21532.67	-469.19		
City of Rocks National Reserve	3rd-order polynomial	45.5	<0.0001	0.92	1300.08	139.08	106.89	-4.44
Clara Barton National Historic Site	1st-order polynomial	11.4	0.007	0.49	862.85	34.36		
Colonial National Historical Park	2nd-order polynomial	52.6	<0.0001	0.9	53866.71	23123.59	-399.19	
Colorado National Monument	3rd-order polynomial	55.5	<0.0001	0.94	16904.89	1241.96	53.52	-2.77
Congaree National Park	2nd-order polynomial	15.8	0.0011	0.73	-3057.69	1280.55	-35.99	
Coronado National Memorial	Null				6422.06			
Cowpens National Battlefield	1st-order polynomial	13.5	0.0043	0.53	8151.96	316.25		

Park	Best-fit model	F-value	P-value	adj. R^2	Intercept	Temperature	$Temperature^2$	$Temperature^3$
Crater Lake National Park	2nd-order polynomial	409.7	<0.0001	0.99	6007.69	-622.37	386.52	
Craters of the Moon National Monument	2nd-order polynomial	609.2	<0.0001	0.99	2998.53	569.78	82.00	
Cumberland Gap National Historical Park	2nd-order polynomial	61.7	<0.0001	0.92	23511.03	5674.61	-94.06	
Cumberland Island National Seashore	2nd-order polynomial	3	0.1006	0.27	-7375.75	1063.88	-23.50	
Curecanti National Recreation Area	2nd-order polynomial	714.9	<0.0001	0.99	45632.73	6229.90	347.45	
Cuyahoga Valley National Park	3rd-order polynomial	19.3	0.0005	0.83	120414.40	-4400.39	1104.46	-29.39
Dayton Aviation Heritage National Historical Park	1st-order polynomial	40.3	<0.0001	0.78	1068.18	290.20		
De Soto National Memorial	1st-order polynomial	9.4	0.0119	0.43	41301.15	-968.84		
Death Valley National Park	3rd-order polynomial	5.3	0.0264	0.54	-55171.40	24694.01	-1381.41	23.59
Delaware Water Gap National Recreation Area	1st-order polynomial	151.9	<0.0001	0.93	211318.50	12728.91		
Denali National Park & Preserve	3rd-order polynomial	101.2	<0.0001	0.96	15830.32	4702.54	419.85	11.56
Devils Postpile National Monument	2nd-order polynomial	44.6	<0.0001	0.89	-172.80	-179.13	142.78	
Devils Tower National Monument	2nd-order polynomial	242.7	<0.0001	0.98	-967.74	-18.36	210.39	
Dinosaur National Monument	2nd-order polynomial	243.1	<0.0001	0.98	7858.83	1182.58	119.09	
Dry Tortugas National Park	Null				3400.37			

Park	Best-fit model	F-value	P-value	adj. R^2	Intercept	Temperature	$Temperature^2$	$Temperature^3$
Edgar Allan Poe National Historic Site	1st-order polynomial	9.5	0.0116	0.44	774.92	24.36		
Edison National Historic Site	3rd-order polynomial	5	0.0307	0.52	2886.77	374.33	-40.69	1.19
Effigy Mounds National Monument	1st-order polynomial	15.5	0.0028	0.57	3927.84	388.97		
Eisenhower National Historic Site	1st-order polynomial	92.9	<0.0001	0.89	710.22	622.57		
El Malpais National Monument	2nd-order polynomial	110.5	<0.0001	0.95	3548.79	666.94	-9.30	
El Morro National Monument	2nd-order polynomial	52.5	<0.0001	0.9	2480.52	348.37	-6.01	
Eleanor Roosevelt National Historic Site	1st-order polynomial	31.1	0.0002	0.73	1442.34	343.22		
Everglades National Park	1st-order polynomial	30	0.0003	0.73	278181.30	-8077.70		
Federal Hall National Memorial	3rd-order polynomial	28.1	0.0001	0.88	9498.33	1371.27	-129.26	4.14
Fire Island National Seashore	3rd-order polynomial	131.6	<0.0001	0.97	2683.62	4398.44	-642.95	30.82
Florissant Fossil Beds National Monument	3rd-order polynomial	356	<0.0001	0.99	2765.91	264.54	9.79	1.99
Ford's Theatre National Historic Site	2nd-order polynomial	2.6	0.1256	0.23	5119.85	10188.64	-301.01	
Fort Bowie National Historic Site	3rd-order polynomial	6.7	0.0141	0.61	-1114.79	399.82	-23.68	0.41
Fort Caroline National Memorial	3rd-order polynomial	6	0.0189	0.58	-56554.49	10971.68	-531.68	8.19

Park	Best-fit model	F-value	P-value	adj. R^2	Intercept	Temperature	$Temperature^2$	$Temperature^3$
Fort Davis National Historic Site	3rd-order polynomial	4.3	0.0439	0.47	-16450.21	3917.24	-218.59	3.86
Fort Donelson National Battlefield	1st-order polynomial	74.5	<0.0001	0.87	12591.93	557.09		
Fort Frederica National Monument	3rd-order polynomial	3.1	0.0899	0.36	-134592.00	24280.70	-1196.60	19.17
Fort Laramie National Historic Site	2nd-order polynomial	209.1	<0.0001	0.97	662.92	59.15	29.38	
Fort Larned National Historic Site	1st-order polynomial	100.4	<0.0001	0.9	941.85	181.22		
Fort Matanzas National Monument	1st-order polynomial	11.9	0.0062	0.5	6625.35	1737.40		
Fort McHenry National Monument and Historic Shrine	1st-order polynomial	98.8	<0.0001	0.9	22287.22	2389.99		
Fort Necessity National Battlefield	1st-order polynomial	90.3	<0.0001	0.89	2355.27	936.24		
Fort Point National Historic Site	1st-order polynomial	34	0.0002	0.75	14192.92	7026.51		
Fort Pulaski National Monument	1st-order polynomial	10.9	0.0079	0.47	11240.99	926.38		
Fort Raleigh National Historical Site	2nd-order polynomial	30.8	<0.0001	0.84	25327.78	-4194.64	212.30	
Fort Scott National Historic Site	1st-order polynomial	13.4	0.0044	0.53	547.32	254.11		
Fort Smith National Historic Site	1st-order polynomial	52.6	<0.0001	0.82	1471.85	372.44		
Fort Stanwix National Monument	3rd-order polynomial	13	0.0019	0.77	2757.04	-76.86	-52.08	4.90

Park	Best-fit model	F-value	P-value	adj. R^2	Intercept	Temperature	$Temperature^2$	$Temperature^3$
Fort Sumter National Monument	1st-order polynomial	13.4	0.0044	0.53	2530.45	2003.87		
Fort Union National Monument	3rd-order polynomial	59.9	<0.0001	0.94	398.38	134.11	-10.68	0.45
Fort Union Trading Post National Historic Site	2nd-order polynomial	31.5	<0.0001	0.85	185.55	74.41	7.00	
Fort Vancouver National Historic Site	1st-order polynomial	17.3	0.002	0.6	3137.88	2974.62		
Fort Washington Park	2nd-order polynomial	27.3	0.0002	0.83	4479.45	2374.20	-40.92	
Fossil Butte National Monument	2nd-order polynomial	286.4	<0.0001	0.98	282.89	93.48	9.91	
Franklin Delano Roosevelt Memorial National Memorial	2nd-order polynomial	5.4	0.0282	0.45	-48362.64	46016.33	-1366.65	
Frederick Douglass National Historic Site	1st-order polynomial	3.5	0.0914	0.18	2133.63	89.79		
Fredericksburg and Spotsylvania National Military Park	1st-order polynomial	68.4	<0.0001	0.86	29148.53	1296.36		
Friendship Hill National Historic Site	3rd-order polynomial	6.4	0.0163	0.59	528.94	-196.59	58.86	-2.13
Gateway National Recreation Area	3rd-order polynomial	292.3	<0.0001	0.99	147554.40	48135.67	-4752.53	211.26
Gauley River National Recreation Area	1st-order polynomial	2.9	0.1189	0.15	3319.27	933.70		
General Grant National Memorial	3rd-order polynomial	15.5	0.0011	0.8	1821.07	1145.40	-98.05	2.76
George Rogers Clark National Historical Park	1st-order polynomial	4	0.0731	0.21	2520.12	614.93		

Park	Best-fit model	F-value	P-value	adj. R^2	Intercept	Temperature	$Temperature^2$	$Temperature^3$
George Washington Birthplace National Monument	1st-order polynomial	58.1	<0.0001	0.84	2356.60	583.35		
George Washington Carver National Monument	1st-order polynomial	61.1	<0.0001	0.85	975.55	240.09		
George Washington Memorial Parkway	2nd-order polynomial	24.4	0.0002	0.81	191394.60	42858.54	-782.54	
Gettysburg National Military Park	1st-order polynomial	90.6	<0.0001	0.89	27237.60	7737.48		
Gila Cliff Dwellings National Monument	3rd-order polynomial	19.8	0.0005	0.84	-138.28	1071.69	-101.65	3.25
Glacier Bay National Park & Preserve	3rd-order polynomial	109.6	<0.0001	0.97	2650.24	4702.55	519.27	-33.01
Glacier National Park	3rd-order polynomial	960.7	<0.0001	0.99	28247.20	9302.49	1557.36	85.79
Glen Canyon National Recreation Area	2nd-order polynomial	226.6	<0.0001	0.98	51154.33	6791.90	326.95	
Golden Gate National Recreation Area	1st-order polynomial	97	<0.0001	0.9	396508.70	62916.37		
Golden Spike National Historic Site	1st-order polynomial	36.7	0.0001	0.76	1124.24	297.10		
Governors Island National Monument	2nd-order polynomial	77.5	<0.0001	0.93	570.70	-1277.54	140.21	
Grand Canyon National Park	1st-order polynomial	324.3	<0.0001	0.97	66033.24	19579.11		
Grand Portage National Monument	3rd-order polynomial	32.2	<0.0001	0.89	860.54	15.01	20.12	1.83
Grand Teton National Park	2nd-order polynomial	376.6	<0.0001	0.99	40894.46	12233.94	1487.52	

Park	Best-fit model	F-value	P-value	adj. R^2	Intercept	Temperature	Temperature^2	Temperature^3
Grant-Kohrs Ranch National Historic Site	2nd-order polynomial	647.3	<0.0001	0.99	308.05	49.95	12.22	
Great Basin National Park	2nd-order polynomial	374.2	<0.0001	0.99	1826.10	407.67	10.18	
Great Sand Dunes National Park	2nd-order polynomial	93.7	<0.0001	0.94	4719.53	901.68	94.56	
Great Smoky Mountains National Park	1st-order polynomial	57.8	<0.0001	0.84	144206.50	46971.25		
Greenbelt Park	1st-order polynomial	116.5	<0.0001	0.91	6530.74	1006.24		
Guadalupe Mountains National Park	3rd-order polynomial	9.8	0.0047	0.71	-24798.29	7641.73	-432.30	7.64
Guilford Courthouse National Military Park	3rd-order polynomial	17.9	0.0007	0.82	6747.23	4545.27	-273.81	5.53
Gulf Islands National Seashore	1st-order polynomial	35.7	0.0001	0.76	-64558.59	21423.26		
Hagerman Fossil Beds National Monument	1st-order polynomial	42.2	<0.0001	0.79	287.75	117.78		
Haleakala National Park	1st-order polynomial	21.5	0.0009	0.65	-41045.18	6395.62		
Hamilton Grange National Memorial	2nd-order polynomial	18.7	0.0006	0.76	1164.88	136.73	-4.83	
Hampton National Historic Site	1st-order polynomial	3.1	0.1093	0.16	2297.19	61.27		
Harpers Ferry National Historical Park	2nd-order polynomial	28.6	0.0001	0.83	694.47	4084.69	-72.01	
Harry S. Truman National Historic Site	2nd-order polynomial	160.6	<0.0001	0.97	1838.82	164.73	5.25	

Park	Best-fit model	F-value	P-value	adj. R^2	Intercept	Temperature	$Temperature^2$	$Temperature^3$
Hawai'i Volcanoes National Park	2nd-order polynomial	6.6	0.0175	0.5	2209443.00	-214686.60	5475.40	
Herbert Hoover National Historic Site	2nd-order polynomial	35.1	<0.0001	0.86	10995.88	150.25	23.91	
Home of Franklin D. Roosevelt National Historic Site	1st-order polynomial	42.8	<0.0001	0.79	5460.35	844.45		
Homestead National Monument	1st-order polynomial	36.2	0.0001	0.76	1731.81	174.88		
Hopewell Culture National Historical Park	1st-order polynomial	92.2	<0.0001	0.89	529.17	234.06		
Hopewell Furnace National Historic Site	1st-order polynomial	172.3	<0.0001	0.94	3584.51	391.16		
Horseshoe Bend National Military Park	2nd-order polynomial	9.8	0.0056	0.61	-1135.05	818.16	-18.62	
Hot Springs National Park	1st-order polynomial	153.4	<0.0001	0.93	35489.01	4389.09		
Hovenweep National Monument	3rd-order polynomial	68.1	<0.0001	0.95	291.91	110.82	10.74	-0.42
Hubbell Trading Post National Historic Site	3rd-order polynomial	74.9	<0.0001	0.95	5847.24	450.32	55.83	-2.29
Independence National Historical Park	1st-order polynomial	70.1	<0.0001	0.86	124859.90	13793.50		
Indiana Dunes National Lakeshore	3rd-order polynomial	142.1	<0.0001	0.97	67939.05	4182.25	-161.58	19.26
Isle Royale National Park	3rd-order polynomial	88.3	<0.0001	0.96	-116.80	-73.79	6.84	1.01
James A Garfield National Historic Site	2nd-order polynomial	28.2	0.0001	0.83	822.99	28.43	3.81	

Park	Best-fit model	F-value	P-value	adj. R^2	Intercept	Temperature	Temperature^2	Temperature^3
Jean Lafitte National Historical Park and Preserve	Null				55256.64			
Jefferson National Expansion Memorial National Memorial	3rd-order polynomial	13.5	0.0017	0.77	10449.77	48054.09	-4887.69	144.38
Jewel Cave National Monument	3rd-order polynomial	205.5	<0.0001	0.98	534.39	39.78	19.85	3.70
Jimmy Carter National Historic Site	2nd-order polynomial	8.5	0.0085	0.58	-5391.05	1196.59	-31.46	
John D. Rockefeller, Jr. Memorial Parkway	2nd-order polynomial	477	<0.0001	0.99	9930.38	7464.59	959.52	
John Day Fossil Beds National Monument	1st-order polynomial	273.9	<0.0001	0.96	2407.82	922.37		
John F. Kennedy Center for Performing Arts	2nd-order polynomial	3.9	0.0605	0.34	201747.60	14617.79	-430.51	
John Fitzgerald Kennedy National Historic Site	1st-order polynomial	84.4	<0.0001	0.88	336.80	86.21		
John Muir National Historic Site	2nd-order polynomial	3.7	0.0674	0.33	-9288.97	1632.34	-54.43	
Johnstown Flood National Memorial	1st-order polynomial	246.7	<0.0001	0.96	3821.07	551.25		
Joshua Tree National Park	3rd-order polynomial	8.6	0.0069	0.67	-471694.20	93487.53	-4537.80	66.80
Kalaupapa National Historical Park	Null				5111.93			
Kaloko-Honokohau National Historical Park	Null				6453.96			
Katmai National Park & Preserve	3rd-order polynomial	51.4	<0.0001	0.93	357.25	-11.47	41.87	6.13
Kenai Fjords National Park	2nd-order polynomial	299.2	<0.0001	0.98	1062.40	2021.56	310.83	

Park	Best-fit model	F-value	P-value	adj. R^2	Intercept	Temperature	$Temperature^2$	$Temperature^3$
Kennesaw Mountain National Battlefield Park	2nd-order polynomial	19.9	0.0005	0.77	-2558.84	10755.81	-251.53	
Kings Canyon National Park	2nd-order polynomial	411.7	<0.0001	0.99	21020.27	3102.06	208.79	
Kings Mountain National Military Park	2nd-order polynomial	82.1	<0.0001	0.94	3324.02	1808.79	-38.79	
Klondike Gold Rush National Historical Park (AK)	2nd-order polynomial	172.4	<0.0001	0.97	24569.82	7303.35	438.60	
Klondike Gold Rush National Historical Park (WA)	3rd-order polynomial	215.1	<0.0001	0.98	-603.83	1111.94	-93.01	3.67
Knife River Indian Villages National Historic Site	2nd-order polynomial	163.4	<0.0001	0.97	358.19	84.68	5.65	
Korean War Veterans Memorial National Memorial	2nd-order polynomial	9.2	0.0067	0.6	-4557.37	36624.43	-995.61	
Lake Chelan National Recreation Area	3rd-order polynomial	1249.3	<0.0001	0.99	1305.70	233.39	22.00	0.47
Lake Clark National Park & Preserve	2nd-order polynomial	28.2	0.0001	0.83	506.08	97.16	6.57	
Lake Mead National Recreation Area	2nd-order polynomial	32	<0.0001	0.85	170250.60	39729.03	-594.82	
Lake Meredith National Recreation Area	1st-order polynomial	37.9	0.0001	0.77	21175.32	6128.08		
Lake Roosevelt National Recreation Area	3rd-order polynomial	592	<0.0001	0.99	32991.06	2798.27	-328.22	43.04
Lassen Volcanic National Park	2nd-order polynomial	159	<0.0001	0.97	14733.12	-2645.39	344.09	
Lava Beds National Monument	2nd-order polynomial	247.3	<0.0001	0.98	4074.80	282.53	19.89	

Park	Best-fit model	F-value	P-value	adj. R^2	Intercept	Temperature	$Temperature^2$	$Temperature^3$
Lewis and Clark National Historical Trail	3rd-order polynomial	41.8	<0.0001	0.92	-64863.48	22666.80	-2292.80	81.13
Lincoln Boyhood National Memorial	2nd-order polynomial	61	<0.0001	0.92	2570.69	166.49	36.81	
Lincoln Home National Historic Site	1st-order polynomial	47.6	<0.0001	0.81	10600.42	2011.96		
Lincoln Memorial National Memorial	2nd-order polynomial	14.4	0.0016	0.71	48543.54	28547.64	-612.43	
Little Bighorn Battlefield National Monument	2nd-order polynomial	237.8	<0.0001	0.98	1841.75	575.09	131.65	
Little River Canyon National Preserve	1st-order polynomial	42.1	<0.0001	0.79	3399.28	836.22		
Little Rock Central High School National Historic Site	3rd-order polynomial	8.2	0.0079	0.66	-3041.04	1212.27	-63.19	1.07
Longfellow National Historic Site	1st-order polynomial	132.8	<0.0001	0.92	538.69	160.40		
Lowell National Historical Park	3rd-order polynomial	6.5	0.0151	0.6	34272.78	2940.09	-931.22	47.10
Lyndon B. Johnson National Historic Park	2nd-order polynomial	2.5	0.1354	0.22	-11098.04	2905.79	-71.44	
Lyndon Baines Johnson Memorial Grove on the Potomac National Memorial	1st-order polynomial	60.1	<0.0001	0.84	34238.86	1095.39		
Maggie L. Walker National Historic Site National Historic Site	Null				679.21			
Mammoth Cave National Park	3rd-order polynomial	73.1	<0.0001	0.95	24799.62	14230.44	-1060.78	32.49
Manassas National Battlefield Park	2nd-order polynomial	13	0.0022	0.69	10768.06	6343.07	-141.97	

Park	Best-fit model	F-value	P-value	adj. R^2	Intercept	Temperature	$Temperature^2$	$Temperature^3$
Manzanar National Historic Site	3rd-order polynomial	29.4	0.0001	0.89	2307.58	1166.23	-114.26	4.06
Marsh-Billings-Rockefeller National Historical Park	1st-order polynomial	9.5	0.0116	0.44	1770.37	150.30		
Martin Luther King, Jr. National Historic Site	2nd-order polynomial	9.8	0.0055	0.62	171511.00	-18466.55	612.06	
Martin Van Buren National Historic Site	1st-order polynomial	22.7	0.0008	0.66	260.61	105.13		
Mary McLeod Bethune Council House National Historic Site	Null				870.31			
Mesa Verde National Park	2nd-order polynomial	737.2	<0.0001	0.99	5430.68	887.89	233.47	
Minute Man National Historical Park	2nd-order polynomial	43.4	<0.0001	0.89	41065.69	6533.23	-128.05	
Minuteman Missile National Historic Site	2nd-order polynomial	109.9	<0.0001	0.95	-56.84	4.33	16.94	
Missouri National Recreation River	2nd-order polynomial	175.1	<0.0001	0.97	4530.40	404.62	18.42	
Mojave National Preserve	2nd-order polynomial	3.2	0.0915	0.28	23774.24	2717.06	-75.46	
Monocacy National Battlefield	1st-order polynomial	93.6	<0.0001	0.89	944.47	67.04		
Montezuma Castle National Monument	3rd-order polynomial	7.5	0.0105	0.64	-21385.71	14662.27	-786.67	13.36
Moores Creek National Battlefield	1st-order polynomial	9.3	0.0122	0.43	2663.77	121.64		
Morristown National Historical Park	2nd-order polynomial	63.7	<0.0001	0.92	25682.30	2340.06	-57.50	

Park	Best-fit model	F-value	P-value	adj. R^2	Intercept	Temperature	$Temperature^2$	$Temperature^3$
Mount Rainier National Park	2nd-order polynomial	293.5	<0.0001	0.98	28617.90	-2907.66	1265.83	
Mount Rushmore National Memorial	3rd-order polynomial	283.5	<0.0001	0.99	16974.62	947.22	392.19	22.90
Muir Woods National Monument	1st-order polynomial	55.4	<0.0001	0.83	-49191.81	10244.96		
Natchez National Historical Park	2nd-order polynomial	7.9	0.0106	0.56	-26166.54	4506.22	-108.05	
Natchez Trace Parkway and National Scenic Trail	2nd-order polynomial	18.9	0.0006	0.76	505242.90	13033.37	-265.39	
National Capital combined Park	1st-order polynomial	34	0.0002	0.75	105910.80	22592.40		
Natural Bridges National Monument	3rd-order polynomial	102.6	<0.0001	0.97	1459.30	459.90	77.98	-3.43
Navajo National Monument	1st-order polynomial	394.9	<0.0001	0.97	1692.80	409.24		
New Bedford Whaling National Historical Park	3rd-order polynomial	3.6	0.0661	0.41	850.00	8065.67	-1137.05	40.18
New River Gorge National River	2nd-order polynomial	58	<0.0001	0.91	18983.62	1078.17	230.95	
Nez Perce National Historical Park	2nd-order polynomial	95.7	<0.0001	0.95	10516.51	539.66	19.92	
Nicodemus National Historic Site	2nd-order polynomial	13.7	0.0019	0.7	764.55	-19.56	4.16	
Ninety Six National Historic Site	2nd-order polynomial	17.9	0.0007	0.75	-859.13	528.15	-13.95	
Niobrara National Scenic River	3rd-order polynomial	89.8	<0.0001	0.96	1512.62	-61.67	-38.55	3.29

Park	Best-fit model	F-value	P-value	adj. R^2	Intercept	Temperature	Temperature^2	Temperature^3
North Cascades National Park	2nd-order polynomial	211.8	<0.0001	0.97	2881.44	1373.89	264.31	
Obed Wild and Scenic River	1st-order polynomial	25.5	0.0005	0.69	6391.28	669.28		
Ocmulgee National Monument	2nd-order polynomial	3.8	0.065	0.33	-5482.38	1897.01	-49.09	
Olympic National Park	3rd-order polynomial	68.9	<0.0001	0.95	-169292.10	113264.60	-14829.97	727.70
Oregon Caves National Monument	3rd-order polynomial	131.6	<0.0001	0.97	-2420.99	1437.30	-139.85	6.92
Organ Pipe Cactus National Monument	1st-order polynomial	10.5	0.0088	0.46	44654.17	-1082.33		
Ozark National Scenic Riverway	3rd-order polynomial	272.4	<0.0001	0.99	6740.23	10841.44	-871.05	34.79
Padre Island National Seashore	1st-order polynomial	25.8	0.0005	0.69	-28204.52	3697.80		
Palo Alto Battlefield National Historic Site	2nd-order polynomial	15.9	0.0011	0.73	-3733.34	708.90	-17.76	
Pea Ridge National Military Park	2nd-order polynomial	27.6	0.0001	0.83	-40.38	785.03	-13.06	
Pecos National Historic Park	1st-order polynomial	244.3	<0.0001	0.96	1621.03	229.23		
Pennsylvania Avenue National Historic Site National Historic Site	1st-order polynomial	21.8	0.0009	0.65	6661.81	870.30		
Perry's Victory and International Peace Memorial	3rd-order polynomial	163.2	<0.0001	0.98	760.26	38.91	-59.22	6.55
Petersburg National Battlefield	1st-order polynomial	15.4	0.0028	0.57	12756.70	763.45		

Park	Best-fit model	F-value	P-value	adj. R^2	Intercept	Temperature	Temperature^2	Temperature^3
Petrified Forest National Park	2nd-order polynomial	43.6	<0.0001	0.89	20787.51	1137.54	110.81	
Petroglyph National Monument	2nd-order polynomial	11.6	0.0032	0.66	3837.69	737.35	-24.70	
Pictured Rocks National Lakeshore	3rd-order polynomial	34.6	<0.0001	0.9	11391.46	-235.69	109.79	9.14
Pinnacles National Monument	2nd-order polynomial	2.7	0.1232	0.23	-39492.22	8825.81	-329.06	
Pipe Spring National Monument	3rd-order polynomial	65.2	<0.0001	0.95	1197.15	165.61	16.43	-0.66
Pipestone National Monument	3rd-order polynomial	137.6	<0.0001	0.97	2146.47	83.71	5.82	1.70
Piscataway Park	2nd-order polynomial	54.7	<0.0001	0.91	2771.23	1414.03	-30.46	
Point Reyes National Seashore	1st-order polynomial	36.7	0.0001	0.76	65674.33	7813.43		
President's Park	1st-order polynomial	10	0.0101	0.45	45860.88	2078.71		
Prince William Forest Park	2nd-order polynomial	41.1	<0.0001	0.88	4009.86	2371.86	-43.13	
Pu'uhonua o Honaunau National Historical Park	Null				36108.03			
Puukohola Heiau National Historic Site	Null				5686.58			
Rainbow Bridge National Monument	3rd-order polynomial	173.5	<0.0001	0.98	1126.07	-277.08	121.99	-2.41
Redwood National Park	3rd-order polynomial	191.8	<0.0001	0.98	-41991.12	16526.90	-1569.76	58.50
Richmond National Battlefield Park	2nd-order polynomial	23.5	0.0003	0.8	4178.22	1405.91	-29.69	

Park	Best-fit model	F-value	P-value	adj. R^2	Intercept	Temperature	$Temperature^2$	$Temperature^3$
Rock Creek Park Park	2nd-order polynomial	362.1	<0.0001	0.98	88326.53	2715.64	126.89	
Rocky Mountain National Park	2nd-order polynomial	220.9	<0.0001	0.98	100771.10	19179.80	1706.51	
Roger Williams National Memorial	3rd-order polynomial	105.2	<0.0001	0.97	974.90	24.89	38.97	-1.42
Ross Lake National Recreation Area	2nd-order polynomial	267.7	<0.0001	0.98	6130.08	2598.83	355.45	
Russell Cave National Monument	2nd-order polynomial	42	<0.0001	0.88	-501.66	292.18	-6.38	
Sagamore Hill National Historic Site	3rd-order polynomial	41	<0.0001	0.92	2267.62	883.81	-62.25	1.76
Saguaro National Park	1st-order polynomial	9.7	0.011	0.44	93178.91	-1882.39		
Saint Croix National Scenic River	3rd-order polynomial	173.3	<0.0001	0.98	5643.28	500.79	67.73	5.97
Saint Paul's Church National Historic Site	Null				882.16			
Saint-Gaudens National Historic Site	2nd-order polynomial	28.1	0.0001	0.83	300.01	113.98	13.14	
Salem Maritime National Historic Site	1st-order polynomial	16.9	0.0021	0.59	19370.98	4000.82		
Salinas Pueblo Missions National Monument	2nd-order polynomial	70.1	<0.0001	0.93	1015.77	311.03	-8.52	
San Antonio Missions National Historic Park	1st-order polynomial	24.5	0.0006	0.68	35385.09	1859.97		
San Francisco Maritime National Historical Park	1st-order polynomial	13.5	0.0042	0.53	-59807.10	26138.46		

Park	Best-fit model	F-value	P-value	adj. R^2	Intercept	Temperature	Temperature^2	Temperature^3
San Juan Island National Historical Park	Null				122752.20			
San Juan National Historical Site	3rd-order polynomial	12.7	0.0021	0.76	##########	2320576.00	-101316.30	1474.31
Santa Monica Mountains National Recreation Area	2nd-order polynomial	2.9	0.1079	0.25	-246855.00	38866.54	-1250.72	
Saratoga National Historical Park	2nd-order polynomial	75.4	<0.0001	0.93	4185.60	500.07	12.41	
Saugus Iron Works National Historic Site	2nd-order polynomial	191	<0.0001	0.97	780.18	113.06	2.71	
Scotts Bluff National Monument	2nd-order polynomial	120.2	<0.0001	0.96	4014.47	292.71	28.60	
Sequoia National Park	2nd-order polynomial	157.1	<0.0001	0.97	22717.88	2173.63	339.01	
Shenandoah National Park	1st-order polynomial	16.9	0.0021	0.59	20097.59	8449.43		
Shiloh National Battlefield	2nd-order polynomial	17.8	0.0007	0.75	4167.83	2901.51	-64.99	
Sitka National Historical Park	2nd-order polynomial	127.9	<0.0001	0.96	3951.18	242.46	279.96	
Sleeping Bear Dunes National Lakeshore	3rd-order polynomial	43.5	<0.0001	0.92	26645.08	-1963.04	-399.82	62.93
Springfield Armory National Historic Site	2nd-order polynomial	35.9	<0.0001	0.86	996.41	24.26	1.94	
Statue Of Liberty National Monument	3rd-order polynomial	58.8	<0.0001	0.94	100745.00	27548.07	-1894.20	58.28
Steamtown National Historic Site	1st-order polynomial	29.4	0.0003	0.72	3883.19	706.67		

Park	Best-fit model	F-value	P-value	adj. R^2	Intercept	Temperature	$Temperature^2$	$Temperature^3$
Stones River National Battlefield	2nd-order polynomial	25.6	0.0002	0.82	5624.22	1069.82	-19.32	
Sunset Crater Volcano National Monument	2nd-order polynomial	89	<0.0001	0.94	10368.14	2231.34	-33.31	
Tallgrass Prairie National Preserve	3rd-order polynomial	20.7	0.0004	0.84	398.96	-75.92	21.40	-0.59
Theodore Roosevelt Birthplace National Historic Site	2nd-order polynomial	5.9	0.023	0.47	1112.23	61.08	-2.51	
Theodore Roosevelt Inaugural National Historic Site	Null				1692.20			
Theodore Roosevelt Island National Memorial	2nd-order polynomial	432.2	<0.0001	0.99	3662.57	429.68	-4.94	
Theodore Roosevelt National Park	3rd-order polynomial	257	<0.0001	0.99	11211.54	1133.72	66.74	5.84
Thomas Jefferson Memorial National Memorial	2nd-order polynomial	5.1	0.0323	0.43	1583.99	19128.95	-467.31	
Timpanogos Cave National Monument	2nd-order polynomial	244.1	<0.0001	0.98	-518.15	-229.99	73.17	
Timucaun Ecological and Historical Preserve Ecological and Historical Preserve	1st-order polynomial	10.7	0.0085	0.47	27915.40	1323.47		
Tonto National Monument	1st-order polynomial	6.6	0.0276	0.34	9481.79	-221.74		
Tumacacori National Historical Park	1st-order polynomial	22.5	0.0008	0.66	9918.25	-320.34		
Tuskegee Airmen National Historic Site	Null				2180.07			
Tuskegee Institute National Historical Site	Null				12939.95			

Park	Best-fit model	F-value	P-value	adj. R^2	Intercept	Temperature	$Temperature^2$	$Temperature^3$
Tuzigoot National Monument	3rd-order polynomial	7.1	0.0119	0.63	-8654.92	3520.36	-199.79	3.48
Ulysses S. Grant National Historic Site	2nd-order polynomial	152.3	<0.0001	0.96	442.16	51.84	2.54	
Upper Delaware Scenic and Recreational River	3rd-order polynomial	141	<0.0001	0.97	1659.23	-63.65	27.93	5.47
Valley Forge National Historical Park	1st-order polynomial	75.9	<0.0001	0.87	116206.40	6056.73		
Vanderbilt Mansion National Historic Site	3rd-order polynomial	44.4	<0.0001	0.92	13425.03	1840.06	107.32	-5.32
Vicksburg National Military Park	2nd-order polynomial	9.7	0.0057	0.61	-19891.54	8036.45	-160.14	
Vietnam Veterans Memorial National Memorial	2nd-order polynomial	10.4	0.0046	0.63	4444.48	37182.40	-954.45	
Virgin Islands National Park	1st-order polynomial	19.9	0.0012	0.63	326478.90	-10588.91		
Voyageurs National Park	2nd-order polynomial	97.9	<0.0001	0.95	3210.53	753.12	88.28	
Walnut Canyon National Monument	2nd-order polynomial	42.6	<0.0001	0.88	3387.90	963.03	-17.92	
War in the Pacific National Historical Park	1st-order polynomial	2.5	0.1418	0.12	66259.10	-2014.50		
Washita Battlefield National Historic Site	2nd-order polynomial	28.9	0.0001	0.84	215.26	86.29	-1.53	
Weir Farm National Historic Site	2nd-order polynomial	37.9	<0.0001	0.87	542.66	94.57	-1.91	
Whiskeytown National Recreation Area	1st-order polynomial	60.9	<0.0001	0.84	-13713.15	8766.17		

Park	Best-fit model	F-value	P-value	adj. R^2	Intercept	Temperature	$Temperature^2$	$Temperature^3$
White Sands National Monument	1st-order polynomial	12.6	0.0052	0.51	21297.28	1387.50		
Whitman Mission National Historic Site	2nd-order polynomial	23.7	0.0003	0.8	969.67	774.31	-17.60	
William Howard Taft National Historic Site	1st-order polynomial	17.3	0.002	0.6	647.61	14.25		
Wilson's Creek National Battlefield	1st-order polynomial	97.2	<0.0001	0.9	5287.34	670.60		
Wind Cave National Park	3rd-order polynomial	282.9	<0.0001	0.99	13630.94	591.82	88.55	6.85
Wolf Trap National Park for the Performing Arts	3rd-order polynomial	369.2	<0.0001	0.99	-4907.44	4526.01	-708.71	30.09
Women's Rights National Historical Park	3rd-order polynomial	34.8	<0.0001	0.9	1183.69	115.37	-22.49	1.21
World War II Memorial National Memorial	2nd-order polynomial	17.4	0.0008	0.75	-36291.07	54315.87	-1405.10	
World War II Valor in the Pacific National Monument National Monument	3rd-order polynomial	6.9	0.0134	0.61	##########	210087.00	-11708.34	215.86
Wrangell-St. Elias National Park & Preserve	2nd-order polynomial	56.2	<0.0001	0.91	3706.63	737.59	32.15	
Wright Brothers National Memorial	3rd-order polynomial	54.6	<0.0001	0.94	-47611.92	13256.05	-843.65	20.25
Wupatki National Monument	2nd-order polynomial	133.6	<0.0001	0.96	3073.87	1655.21	-22.72	
Yellowstone National Park	2nd-order polynomial	513.5	<0.0001	0.99	39434.98	16394.33	2024.93	
Yosemite National Park	2nd-order polynomial	568.9	<0.0001	0.99	78160.87	19801.66	303.27	

Park	Best-fit model	F-value	P-value	adj. R^2	Intercept	Temperature	$Temperature^2$	$Temperature^3$
Zion National Park	2nd-order polynomial	314.5	<0.0001	0.98	39992.28	16627.52	-170.31	
